# Badland landscape response to individual geomorphic events

**DOI:** 10.1038/s41467-021-24903-1

**Published:** 2021-07-30

**Authors:** Ci-Jian Yang, Jens M. Turowski, Niels Hovius, Jiun-Chuan Lin, Kuo-Jen Chang

**Affiliations:** 1 Department of Geography, National Taiwan University, Taipei, Taiwan (R.O.C.); 2 German Research Centre for Geosciences (GFZ), Potsdam, Germany; 3 Institute of Geosciences, University of Potsdam, Potsdam, Germany; 4 Department of Civil Engineering, National Taipei University of Technology, Taipei, Taiwan (R.O.C.)

**Keywords:** Geodynamics, Geomorphology, Geodynamics, Geomorphology

## Abstract

Landscapes form by the erosion and deposition of sediment, driven by tectonic and climatic forcing. The principal geomorphic processes of badland – landsliding, debris flow and runoff erosion – are similar to those in full scale mountain topography, but operate faster. Here, we show that in the badlands of SW Taiwan, individual rainfall events cause quantifiable landscape change, distinct for the type of rainfall. Typhoon rain reduced hillslope gradients, while lower-intensity precipitation either steepened or flattened the landscape, depending on its initial topography. The steep topography observed in our first survey is inconsistent with the effects of any of the rainfall events. We suggest that it is due to the 2016 Mw 6.4 Meinong earthquake. The observed pattern in the badlands was mirrored in the response of the Taiwan mountain topography to typhoon Morakot in 2009, confirming that badlands offer special opportunities to quantify natural landscape dynamics on observational time scales.

## Abstract (web summary)

The relative role of individual forcing events in long-term landscape evolution is challenging to measure in the field. Badlands offer special opportunities to quantify common, natural landscape dynamics on observational time scales.

## Introduction

Landscapes are dynamic systems that evolve under the influence of external drivers, chiefly tectonics and climate. In active mountain belts, earthquakes and rainstorms present the elemental forms of these drivers^1–3^, and their effects nudge landscapes towards a topographic steady state in which the erosion rate is equal to the uplift rate. As a consequence, it is thought that steady-state landscapes record information on the tectonic and climatic history that shaped them^4,5^. For example, channel profiles^6–8^, mountain relief^6,9^, sediment grain size^10^, stratigraphic geometry^10^, and cosmogenic nuclide measurements^11^ have been evaluated with reference to tectonic uplift or precipitation and used as proxies for external tectonic or climatic influences. Nevertheless, the different roles of climate and tectonics in driving landscape evolution are incompletely understood, hindering the effective exploitation of topographic archives. In natural landscapes, the effects of individual events, such as a rainstorm or an earthquake, are often small compared to the scale of the topography, and can rarely be resolved with available measurement techniques. Ultimately, topography reflects the integrated response of the landscape to many different events over the time scales of tectonics and climate, with added complexity due to slow and steady processes and the heterogeneity of the underlying rock mass and vegetation growth. As a result, in most settings, the effects of one particular type of forcing are hard to isolate. Laboratory analogue experiments can achieve this, because they benefit from controled external conditions acting on a simple substrate. However, they suffer from scale dependence of key properties and processes, which makes a direct comparison with natural landscapes difficult^12,13^.

Badland landscapes form in highly erodible, often homogeneous substrates that have the potential to respond measurably to an individual event on spatial and temporal scales that permit direct observation^14,15^. Moreover, they share essential features with full mountain landscapes: they have ridge-and-valley topography with evolving catchments, and host both distributed and concentrated runoff and the mass wasting and channel processes associated with them. Specifically, badlands are prone to landsliding, debris flows, and fluvial erosion and deposition, which are key geomorphic processes in many erosional landscapes. As such, badlands present a simplified natural laboratory for studies of landscape dynamics^16,17^, in which the topographic effects of different types of forcing may be isolated.

**Fig. 1:**
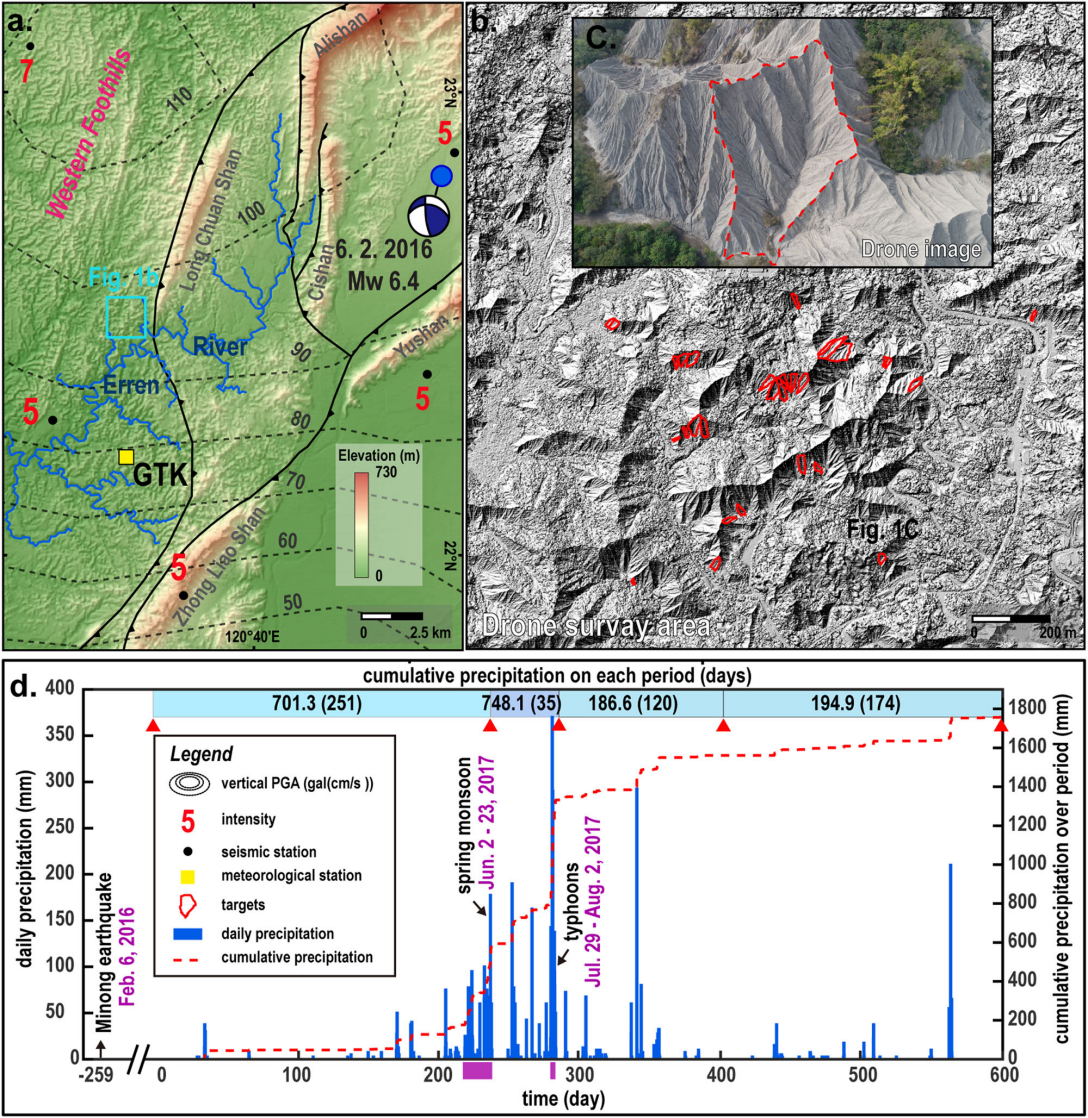
Location and geological background in front of Taiwan orogenic belt. **a** Color-shaded relief map driven from 20 m spatial resolution digital elevation model data (downloaded from https://data.gov.tw/dataset/35430) showing the distribution of major active faults (downloaded from https://www.moeacgs.gov.tw) and intensity of 2016 Meinong earthquakes (red number). Intensity and focal mechanism solution was acquired from the Central Weather Bureau (https://www.cwb.gov.tw/eng/) with a focal mechanism of 295°, 30°, and 37° in a strike, dip, and rake. The black dashed lines denote vertical PGA contours (https://scweb.cwb.gov.tw/special/20160206pga.asp). GTK (yellow square) is a meteorological station providing hourly precipitation data. **b** Shaded relief map of the drone survey area with 30 cm spatial resolution digital surface model data is conducted by this study. The boundaries of target (red block) mapping by this study. **c** Drone image of a typical badland catchment (red dashed block) is conducted by this study. **d** Daily and cumulative precipitation from hourly measurements, red triangles mean UAV survey data.

Mudrock badlands in southwest Taiwan are uplifted rapidly by up to 20 mm/yr^18,19^, and receive rainfall in excess of 2 m/yr^20^. The catchments respond rapidly to this forcing, with catchment-wide denudation rates of 25 mm/yr^2^. Hillslope denudation rates can reach 9–13 cm/yr^21^. This makes it possible to resolve the effects of individual earthquakes and rainstorms on the landscape with current photogrammetric techniques. We observed a representative portion of this landscape (Fig. 1) in four intervals over 19 months, starting 8 months after the 2016 M_w_ 6.4 Meinong earthquake, with mild winter rain, moderate monsoon rain, and two episodes of intense typhoon rain, respectively. These different types of rainfall had distinct effects on the landscape. We focus on the analysis of slope distributions, because the slope is a first-order control on all important erosion processes in the landscape that can be measured everywhere.

Our study area is located in the badland terrain in Tainan County, SW Taiwan (Fig. 1). It has a wet tropical climate with seasonal monsoon rainfall and typhoons. The rainy season lasts from May until October and accounts on average for 89% of the long-term mean annual rainfall of 2118 mm. Average monthly rainfall peaks at >400 mm in August, and is usually <40 mm from November to March. Our study area is located in the footwall of the active Long-Chuan fault, which comprises poorly consolidated mudrocks of the Plio-Pleistocene Gutinkan formation. The area is located in the frontal fold and thrust belt of the Taiwan orogen, which is characterized by frequent, shallow earthquakes^22,23^. The epicentre of the M_W_6.4 Meinong earthquake on 6th February 2016 was located 15 km away from the study area. During this earthquake, peak ground acceleration (PGA) of up to 445 gal was recorded in nearby Tainan city, while PGA was 90–100 gal in the badland study area. Prior to this event, the last earthquake with M_w_ > 6 in the area occurred in 1946.

Following the Meinong earthquake, we surveyed the Tainan badlands five times between October 2016 and May 2018. The four observation periods bracketed by these surveys had three different styles of rainfall, evaluated using hourly precipitation data from Gutingkeng meteorological station (Central Weather Bureau), 5 km from the study area (Fig. 1). Period 1 (22–10, 2016 to 30–06, 2017) comprised the winter dry season and the spring monsoon with 701.3 mm of rainfall over 251 days (maximum intensity of 35.5 mm/hr). Period 2 (01–07 to 04–08, 2017) included typhoons Nesat and Haitang that delivered a total of 748.1 mm of rainfall over 35 days (the typhoons brought 579 mm of rainfall, with a maximum intensity of 74 mm/hr). Periods 3 (05–08 to 02–12, 2017) and 4 (03–12, 2017 to 25–05, 2018) had only minor rainfall events, with a total of 186.6 mm of rainfall during 120 days (maximum intensity of 58.5 mm/hr) and 194.9 mm of rainfall during 174 days (maximum intensity of 42 mm/hr), respectively.

For each interval, we surveyed the same ~1 km^2^ badland area, comprising 30 complete catchments with surface drainage areas ranging from 59.9–1457.5 m^2^, and a total surface area of 0.12 km^2^. This is small relative to the spatial extent of rain events, so that ambient conditions were likely quasi-uniform. The area had an elevation range of 66–143 m asl., a typical valley to ridge relief of 9.0 ± 7.7 m (1 sigma), and an average hillslope gradient of 48.3 ± 9.25 degrees (1 sigma) at the start of our survey period. Due to military control, the catchments were hardly affected by human activity since 1960. The absence of vegetation made digital elevation models (DEM) construction straight-forward. We used photogrammetric Structure from Motion techniques to generate DEM with a pixel size of 30 cm from high-resolution aerial photography obtained by unmanned aerial vehicles and constrained the contribution of survey errors from control points measurement, projection, and image distortion. The main error arises from image distortion with a vertical precision of 0.7–5.6 cm (see Supplementary Table 2). Consecutive DEMs were differenced to estimate local erosion and deposition. We analysed the Spatio-temporal patterns of erosion as a function of the drainage area and the change of hillslope gradient caused by external drivers. The local topographic gradient determines the stability of a site, and the upslope area reflects its position in the landscape and the role concentrated water flow may play in its evolution. Gradient change, calculated for each pixel in each observation period, was binned according to local gradient and upslope area, and median and mean gradient change and its standard deviation were determined for each bin (see Methods).

Here, we study hillslope morphology in the tectonically active, humidific badland area in Taiwan to assess whether the effect of earthquakes and typhoons are reflected in topographic changes obtained from high spatiotemporal resolution digital topographic analysis. We find evidence that earthquakes steepen hillslopes, typhoons decrease hillslope gradients and low-intensity precipitation can either steepen or flatten a landscape. The numerical modeling also demonstrates that the median gradient of hillslope may be used to reflect the driving force. Hillslope gradients as sensitive proxies for landscape change that can provide insights response over much shorter time scales than other topographic features, such as channel long profiles.

## Results and discussion

### Precipitation control on the spatiotemporal distribution of slope erosion

**Fig. 2:**
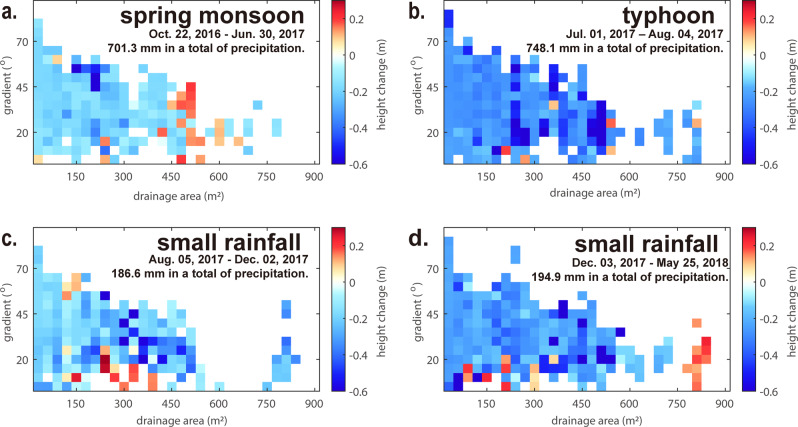
Distribution of mean spatiotemporal erosion for the survey periods. **a** Oct. 22, 2016–Jun. 30, 2017 (monsoon). **b** Jul. 01, 2017–Aug. 04, 2017 (typhoons). **c** Aug. 05, 2017–Dec. 02, 2017 (winter precipitation). **d** Dec. 03, 2017–May 25, 2018 (winter precipitation). The color indicates the mean of height change, warm and cold colors represent positive and negative values, respectively (see Methods).

Over the surveyed periods, about 84% of the study area was affected by erosion. We measured 15 cm of average erosion depth during period 1 with monsoon rainfall, 16 and 18 cm of average erosion depth associated with occasional rainfall in periods 3 and 4, and 28 cm of average erosion depth due to typhoon precipitation in period 2. During monsoon period 1 (Fig. 2a), erosion concentrated at locations with drainage areas of 150–300 m^2^, and deposition concentrated in locations with drainage areas of 450–600 m^2^. During typhoon period 2 (Fig. 2b), erosion was pervasive, with the largest observed erosion rates located on the steepest hillslopes, and at sites with drainage areas of 200–500 m^2^. During periods 3 and 4 without intense precipitation (Fig. 2c, d), erosion concentrated in sites with drainage areas of 300–450 m^2^ and gradients between 20^o^–45^o^.

The described differences and field observations indicate that erosion during the different periods was caused by different geomorphic processes. During monsoon period 1, gully erosion appears to have dominated. During typhoon period 2, gravitational mass wasting preferentially occurred in the steepest locations at any upslope area, including hillslopes (<30 m^2^), gullies (~100 m^2^), and channel walls (>450 m^2^). High erosion rates in channels at large drainage areas during typhoons were likely related to fluvial processes driven by high water discharge. During winter precipitation periods 3 and 4, the observed patterns are indicative of erosion driven by flowing water. In addition, at smaller slopes (<30° of hillslope gradient and 140 m^2^ of the drainage area), where we observed deposition, we found field evidence of mudflows (Supplementary Fig. 2). We infer that water hardly infiltrates the badland hillslopes during minor precipitation events, and rather moves as surface runoff. As a consequence, sediment is mobilized in and transported along gullies where sufficient water can accumulate.

### Topographic steepness and hillslope gradient change

The mean local topographic gradient for the entire surveyed area was 47.24 ± 0.04° (uncertainty gives the standard error of the mean; median 48.3°) in the first survey. During the monsoon period, the balance of erosion at locations with small upslope areas and deposition at locations with large upslope areas caused a small overall decrease in mean hillslope gradient to 46.58 ± 0.04° (47.7°). During the typhoon period, the mean local topographic gradient decreased by 3.31° to 43.27 ± 0.03° (44.2°) due to erosion near the ridges and deposition of eroded sediment downslope, filling many incised channels with steep walls. During the following periods with minor precipitation, the mean hillslope gradient increased by 2.06° to 45.33 ± 0.04° (46.5°), likely due to re-incision and new formation of gullies. The observed differences in mean slopes and the slope distributions of all periods are statistically highly significant (Supplementary Table 3).

**Fig. 3:**
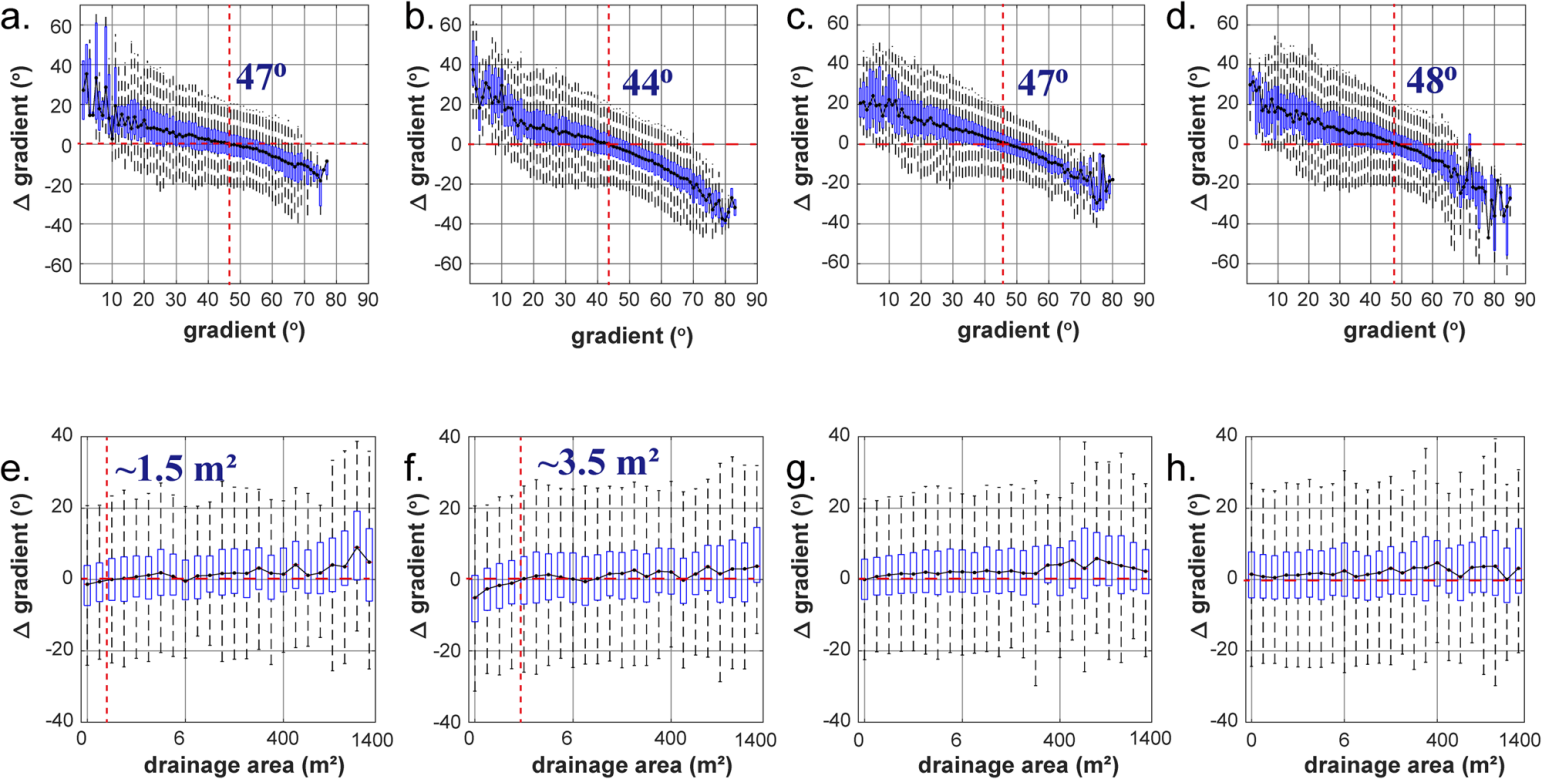
Gradient change as function of hillslope gradient (a–d) and drainage area (e–h) for the four survey periods. Gray dots and error bars denote median and standard deviation of gradient change on each bin, respectively. Horizontal red dashed lines denote no gradient change and vertical red dashed lines denote the inflection of gradient change from steepening to flattening. **a**, **e** Oct. 22, 2016–Jun. 30, 2017 (monsoon). **b**, **f** Jul. 01, 2017–Aug. 04, 2017 (typhoons). **c**, **g** Aug. 05, 2017–Dec. 02, 2017 (winter precipitation). **d**, **h** Dec. 03, 2017–May 25, 2018 (winter precipitation).

All periods show a similar negative, the approximately linear trend in gradient change with increasing gradient (Fig. 3a–d), indicating flattening occurred preferentially in the steepest sites and steepening in sites with lesser gradients. The latter may be related to lateral gully migration observed in all periods (Supplementary Fig. 3), which creates steep erosional channel walls. The transition between flattening and steepening, where the median gradient change is equal to zero, consistently occurred at gradients of 44° to 48° for all observation periods. Additionally, all periods show a weak positive linear trend of median gradient change with increasing drainage area (Fig. 3e–h), indicating that flattening occurred preferentially at small drainage areas (ridges) and steepening at large drainage areas (trunk channels).

### Dynamic equilibrium of hillslopes driven by tectonic and climatic forcing

**Fig. 4:**
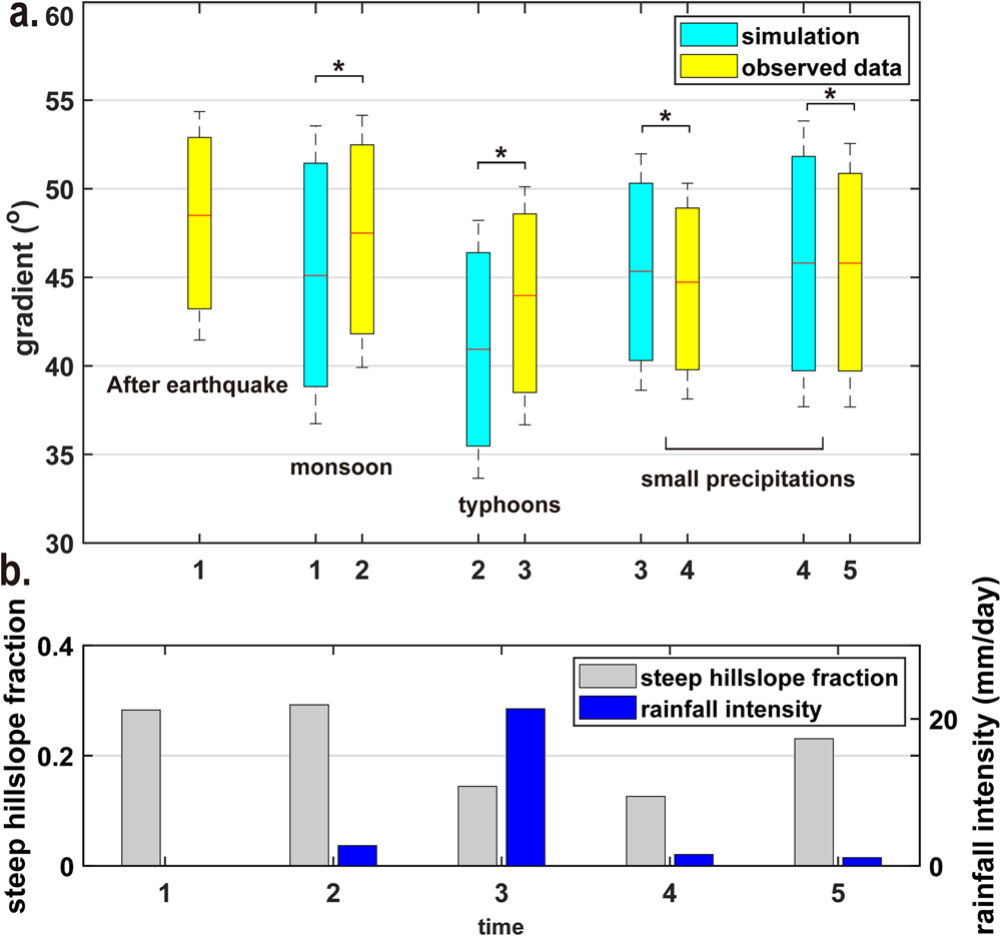
Distribution of hillslope gradients. **a** Compiled observed (yellow) and simulated (cyan) distribution of hillslope gradients. The upper limit of the box means 75% of percentile, the lower limit of the box means 25% of percentile. Significant differences (*p* < 0.01) between the initially observed data and simulations using the Kolmogorov–Smirnov test are indicated by *. **b** Gray bars denote the fraction of steep gradients (> 55^o^) at small drainage areas (<0.5 m^2^). The blue bar denotes rainfall intensity.

To further investigate the dynamics of our study area, we ask how the landscape would respond to each type of forcing (monsoon, typhoon, or occasional rainfall) if only this forcing were active. Because trends with drainage area are weak (Fig. 3e–h), we assume that the response of a particular part of the landscape (i.e., a pixel) depends only on local slope, using the empirical distributions (Fig. 3) to generate random time series of badland slope distributions (Methods). We considered the resulting distribution as the steady-state distribution of gradients in a landscape driven by a single type of precipitation forcing and ask two questions. First, does a steady-state gradient distribution exist for a hypothetical landscape exposed to just one type of precipitation; and second, do all initial gradient configurations evolve towards the same steady-state distribution over time? If both are confirmed, then the differences between observed and modelled steady-state distributions can be used to predict the direction of evolution of hillslope gradients under specific forcing. We assume that the observed distribution of gradient changes associated with a particular type of forcing and its dependence on topographic gradient (Fig. 3a–d) is characteristic, rather than incidental. Starting with a random distribution of topographic gradients and evolving it using distributions fitted to the observed changes (Fig. 3; see Methods), we obtained characteristic modelled steady-state distributions of topographic gradients, for each of the four different survey periods and the associated dominant rainfall type (Fig. 4). These distributions are significantly different for the monsoon, typhoon, and winter precipitation periods. Here, we focus on mean and median slope values, because the variable nature of forcing both in space and in time should affect extreme gradient values more than the central tendencies. The lowest modelled mean gradient, 40.75 ± 0.11° (median 40.9°), occurs for typhoons (Fig. 4). Modelling of monsoon precipitation and winter precipitation resulted in similar steady-state mean values of 45.43 ± 0.12° (45.6°) using constraints from observation period 1, and 45.22 ± 0.10° (45.4°) and 45.7 ± 0.12° (45.9°), using constraints from observation periods 3 and 4, respectively. In comparison, after the monsoon period, the observed mean hillslope gradient of 46.58 ± 0.04° (median 47.7°) was 2.415° higher than the steady-state model value of 45.43 ± 0.12° (45.6°) (Fig. 4a). Similarly, after typhoon impact, the observed value of 43.27 ± 0.03° (45.6°) was 2.5° higher than the model value of 40.75 ± 0.11° (40.9°). This suggests that in these observation periods, not enough time passed to reach a steady-state gradient distribution corresponding to the rainfall events. The median hillslope gradients observed after periods with minor rainfall, 44.24 ± 0.03° (45.5°) and 45.33 ± 0.04° (46.5°) were close to the steady-state model values of 45.22 ± 0.10° (45.4°) and 45.69 ± 0.12° (45.9°) for this type of precipitation.

The observed mean gradient of the first survey was 47.24 ± 0.04° (median 48.3°), higher than in any other period, with a value that unlikely resulted from forcing due to the monsoon, typhoon, or minor precipitation. This implies that another driver has acted to steepen hillslopes beyond the values typical for rainfall forcing. Since anthropogenic influence can be ruled out, we propose that the Meinong earthquake was the likely cause for this steepening. In the 259 days between the earthquake and our first survey, the total rainfall was 1687.5 mm within (maximum intensity of 52 mm/hr), including 572 mm of typhoon rainfall between 25–28 September 2016. If this typhoon precipitation decreased the gradients, as observed for period 2, then the mean and median hillslope gradient likely decreased in the eight months after the earthquake. We expect that immediately after the earthquake, the mean and median hillslope gradients were substantially higher than observed in our first survey. Earthquake-triggered landslides occur preferentially at ridgetops, while precipitation-triggered landslides tend to be located closer to channels^24–26^. Although landslides can act to remove the steepest elements from a landscape, their crests are formed by steep backwalls. Due to this effect, the average gradients of hillslopes at locations with small drainage areas may increase during an earthquake. In agreement with this mechanism, in the topographic data from the first survey, nearly 28% of the hillslope pixels at small drainage areas (<0.5 m^2^) had a slope >55^o^ (Fig. 4b). After period 1 with mild monsoon rain, the fraction of steep pixels at small drainage areas remained about the same at 29%, but after the 2017 typhoons, it reduced to 15%. A further slight decrease to 13% followed during period 3, and then an increase to 23% in our final survey.

### From badland to mountain belt

Our findings show that significant rainfall events and likely single earthquakes can cause sufficient erosion and deposition to measurably change the topography of badland landscapes. While the 2016 Meinong earthquake may have increased the average topographic slope of the Tainan county badlands, intense typhoon rainfall in the summer of 2017 decreased their average topographic gradient. Low-intensity monsoon and winter precipitation either steepened or flattened the landscape, depending on whether the median gradient was below or above the steady-state value for the dominant precipitation type. In our study area, the median local topographic slope of badland catchments after earthquake strong ground motion was >7 degrees steeper than after subsequent periods with very intense precipitation. The observed topographic differences likely arise from systematic differences between types of forcing in the location, the relative importance, and preferential location of geomorphic processes such as landslide, debris flow, and stream incision, and the downslope deposition of their products. This is in agreement with previous notions^24–28^, and allows us to isolate distinct influences of different types of precipitation and of earthquakes on the landscape scale, expressed clearly in the catchment-wide statistics of local topographic slopes.

In full-sized mountain landscapes, steady-state slopes are set by the catchment-wide erosion rate, which is controlled by the rate of tectonic uplift or base level lowering^29^. The physical erosion processes operating in badlands are similar to those in larger-scale steep land and mountain landscapes, and in both, their location depends on a local topographic gradient and upslope area^30–32^. If we accept that badlands can be viewed as a fast-eroding analogue to full-sized mountain landscapes, then extrapolation of our observations suggests that the mean and median slope of mountain landscapes may fluctuate around a steady-state value, which depends on the type of forcing, the sequence of event magnitudes, and the pattern of geomorphic processes. Consequently, we expect that the relationships between the distribution of topographic gradient and gradient change in actively eroding mountain landscapes also show a linearly declining relationship (cf. Fig. 3a–d), albeit with a smaller magnitude of change. Most likely, current survey techniques can resolve this relationship only for the largest forcing events like large earthquakes or exceptional rainstorms, and only within the perimeter of the most intensely impacted area. Typhoon Morakot, one of the most extreme precipitation events ever recorded, hit southern Taiwan in 2009 (Supplementary Fig. 7). This typhoon delivered >2.5 m of precipitation in 2 days and caused widespread land sliding and sediment mobilization^33^. In the upstream part of the Lin-Bian catchment (44 km^2^), gradient change due to typhoon-driven erosion was inversely related to the initial hillslope gradient (Supplementary Fig. 7b), as observed for the badlands (Fig. 3b), leading to significant differences in the slope distributions. Still, even this exceptional event did not cause a measurable change in the median landscape slope. These observations reinforce the special opportunity offered by simple badland landscapes to quantify common, natural landscape dynamics on observational time scales, using simple survey techniques.

## Methods

### Digital elevation model (DEM) created from an unmanned aerial vehicle (UAV)

We used UAV photogrammetry to construct Digital Elevation Models (DEM), which includes imagery acquisition from UAV and image orthorectification using the SfM-MVS photogrammetric techniques. The accuracy of ground control points (GCP) is critically important on achieving precise georeferencing for ortho-images and DEMs. We installed 11 GCPs across the surveying area (1 km^2^) and measured the coordinates of GCPs using Leica RX1250XC differential GPS to an accuracy of 2.5 cm on the vertical axis (Supplementary Table 1). The GCPs are made of 5 mm diameter iron nails surrounded by white paint aerial photogrammetric targets and all GCPs are located on cement roads or bridges, ensuring stability. We measured GPS locations for 15 min at each GCP and used the average values as the resultant coordinate. In sum, the main source of survey error comes from image distortion, reaching tens of centimeters in the horizontal direction, however, the errors in the vertical direction is the level of millimeters to centimeters (Supplementary Table 1 and Supplementary Table 2). Imagery acquisition was carried out using two different UAVs: eBee Classic (surveys 1, 2, and 3) and eBee X (surveys 4 and 5). UAV surveys were acquired 5 times over a period of 20 months between October 2016 and May 2018. The time of acquisition was chosen according to weather conditions. In order to take high-resolution images, we specified a front overlap ratio of 80% and a side overlap ratio of 70%. Further, we limited flight height below 430 m, and thus the ground sample distance (GSD) reached 9.7–11.3 cm/pixel during observed periods. We used the SfM-MVS software by Acute3D (Bentley Systems, Incorporated) to generate 3D meshes, ortho-images, point clouds, DEMs, and relative parameters, e.g., image distortion (see Supplementary Table 1 and Supplementary Fig. 6). In order to avoid shadowing, excessive image distortion, and vegetation effects, we mapped 30 hillslopes as the analysis targets using ortho-images and used DEMs for further analysis.

### Level of detection (LoD) calculation

In order to avoid the imprecision of the instrument and the deviations caused by the co-registration, we calculated the difference of DEM^34,35^ (DoD). DoD denotes the difference between two DEMs of the same area but acquired at two different times^34,35^. In DoDs, the combined errors are as follows:

1 $${{{{{\rm{\delta }}}}}}{{{{{\rm{DoD}}}}}}=\sqrt{{{{{{{\rm{\delta }}}}}}{{{{{\rm{Z}}}}}}}_{1}^{2}+{{{{{{\rm{\delta }}}}}}{{{{{\rm{Z}}}}}}}_{2}^{2}}$$

where δZ_1_ and δZ_2_ represent the standard deviation of the height values of all measurements located within the pixel in question, for the two surveys relevant for the comparison. Since we calculate the standard error of point clouds for every pixel, we obtain a heterogeneous survey error over the entire grid. We used a significance threshold at a critical *t*-value t_crit_ of 1.036 from Student’s t-distribution, corresponding to a confidence level of 70%, below which we assumed no surface change within this area^36^. The level of detection (LoD) was calculated based on the following equation:

2 $${{{{{\rm{LoD}}}}}}={{{{{{\rm{t}}}}}}}_{{{{{{\rm{crit}}}}}}}\,\sqrt{{{{{{{\rm{\delta }}}}}}{{{{{\rm{Z}}}}}}}_{1}^{2}+{{{{{{\rm{\delta }}}}}}{{{{{\rm{Z}}}}}}}_{2}^{2}}$$

With a grid spacing below 11 cm, we found that pixels did not contain enough points for calculating the LoD. As a result, we determined 30 cm to be a suitable grid spacing with an average point density of 15 points per pixel. The calculated LoDs ranged from 7–10 cm. DEM differences with values inside ± LoD and outside ± 2σ of all height changes for a given period were considered as outlier values and were removed from further analysis.

### Spatial erosion calculation and gradient change analysis

In this study, height change is defined as a difference between two DEM values used to interpret erosion. Positive height change means deposition; negative height change means erosion. In order to obtain the hillslope erosion patterns, we calculated hillslope gradient and drainage area at each grid using DEMs constructed from UAV photogrammetry with TopoToolbox 2^37^. Thus, each grid node has an associated height change, and a corresponding gradient and drainage area. We used the two-sample Kolmogorov–Smirnov test and the two-tailed *t*-test to evaluate whether the gradient change distributions and their means measured in the four survey periods are significantly different from each other (Supplementary Table 3). For further analysis, we binned gradient data using bin boundaries every 5 degrees of gradient and every 30 m^2^ of the drainage area and calculated the median of height change on each bin. Because there are few pixels with large drainage area, we limited the analysis to pixels with drainage area below 900 m^2^. Gradient change was defined as a difference of gradient between observations in two surveys in a subsequent period. A positive gradient change means steepening, a negative gradient change means a reduction in gradient. For further analysis, we binned gradient change data using bin boundaries of one degree and 30 m^2^ of drainage area and calculated the median and standard deviation for each bin.

### Steady-state gradient distribution simulations

With the Monte–Carlo model simulations, we explore the consequences of the observed gradient change data (Fig. 3a–d) for the steady-state distribution of slope values, assuming that a single type of precipitation event, for example typhoon rainfall, is driving landscape adjustment. The main assumption was that, for a given slope, the observed distribution of gradient changes is representative of the forcing of the corresponding observation period. We focused on a slope since the drainage area only has a weak control on gradient change distributions (Fig. 3e–h). We created an initial artificial surface on a matrix with 1000 × 1000 nodes and assigned random gradient values between 0 and 90 degrees to each node. We ignored potential spatial and temporal correlations of change and treated each pixel to be independent of each other node. To model the evolution of an individual node, potential changes in gradient are related to the current gradient in this node, using the observed relationships (Fig. 3a–d). Gradient change values were binned using bin boundaries every three degrees from 0 to 90 degrees, to balance a resolution as fine as possible with a sufficient number of data points within each bin to obtain reliable statistics. For all data points within each bin, we fitted the generalized extreme value (GEV) distribution in Matlab2020a to the gradient change values. At each time step, for each node, we used the fitted GEV distribution corresponding to the gradient at the node to randomly sample a gradient change value. We updated the gradient in each node by adding its random gradient change value to its current gradient value. The procedure was repeated until the simulated median gradient of all gradient values in the matrix changed by less than 1 degree. As such, we obtained a modelled steady-state hillslope gradient distribution for each of the four specific precipitation conditions.

### Typhoon Morakot: data sources and analysis

The images before Typhoon Morakot were provided by the Aerial Survey Office, Forestry Bureau, ROC, and were obtained using an aircraft carrier with an Airborne Digital Sensor ADS40 airborne camera. Data acquisition of images after Typhoon Morakot was performed using UAV-Skywalker X8 carry with Nikon D800E with a fixed lens of 50 mm focal length. We selected 38 and 23 checkpoints, respectively, for examining the accuracy of two DEMs. The checkpoint densities spaced from 0.05–0.25 pts/m^2^ and the mean of the height difference between surveyed DSMs and airborne LiDAR is 0.65 m. The standard deviation of the height values of two DEMs are 1.99 and 2.09 m, respectively and therefore the LoD is 2.99 m. We calculated gradients and gradient change for the upstream part of the Lin-Bian catchment (44 km^2^) as has been described above. We used the two-sample Kolmogorov-Smirnov test and the two-tailed t-test to assess whether pre- and post-storm slope distributions and their means are different.

## Supplementary information

Supplementary Info

## Data Availability

All data analysed in this study are available at (https://figshare.com/s/5baddd0080243e5220e1). Other relevant data supporting the findings of the study are available in the Supplementary Information, or from the corresponding author upon request. Source data are provided with this paper.

## Code availability

The MATLAB code used to implement the analysis of the geomorphic data is available on request from the corresponding author.
