# Supplementary material for: Badland landscape response to individual geomorphic events
Source: Nat Commun. 2021 Jul 30;12:4631. doi: 10.1038/s41467-021-24903-1 (PMC8324919; doi:10.1038/s41467-021-24903-1)
Supplement: Supplementary file 1 — Supplementary Info [file 41467_2021_24903_MOESM1_ESM.pdf]

Supplementary Information for

Badland landscape response to individual climatic and tectonic events

Ci-Jian Yang[1,2]✉, Jens M. Turowski[2], Niels Hovius[2,3], Jiun-Chuan Lin[1], Kuo-Jen Chang[4]

1 Department of Geography, National Taiwan University, No. 1, Sec. 4, Roosevelt Rd., Taipei 10617, Taiwan (R.O.C.)

2 German Research Centre for Geosciences (GFZ), Telegrafenberg 14473, Potsdam, Germany

3 Institute of Geosciences, University of Potsdam, Karl Liebknecht Straße 24-25, 14476, Potsdam, Germany

4 Department of Civil Engineering, National Taipei University of Technology, No.1, Sec. 3, Zhongxiao E. Rd., Taipei 10608, Taiwan (R.O.C)

✉: d03228001@ntu.edu.tw

Contents of this File

Supplementary Figure 1–9 and Table. 1–3.

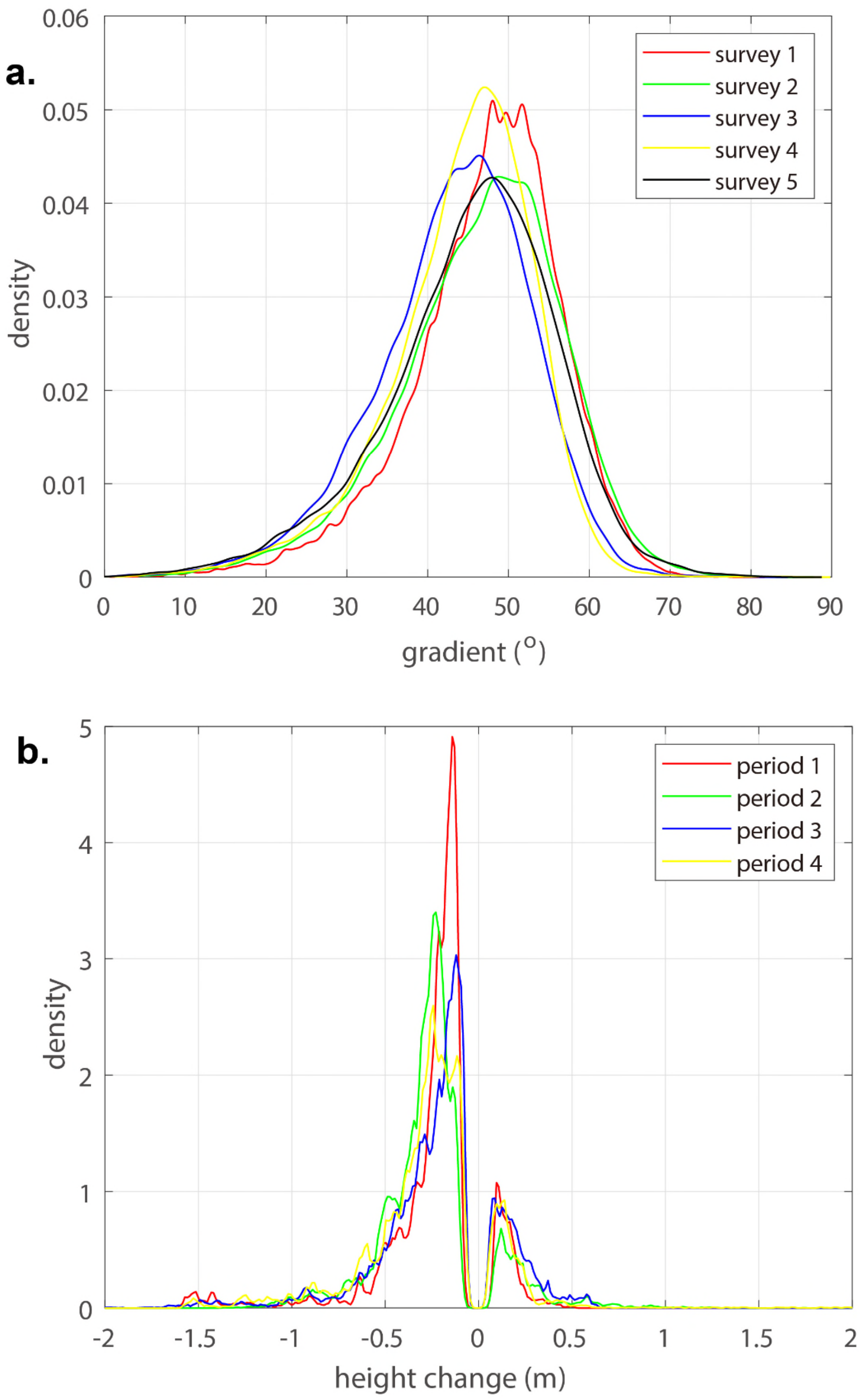

Supplementary Figure 1 kernel density plots of gradient and height change of the all epochs. a distribution of gradient, colors are corresponded to survey date. **b** distribution of height change, colors are corresponded to survey period.

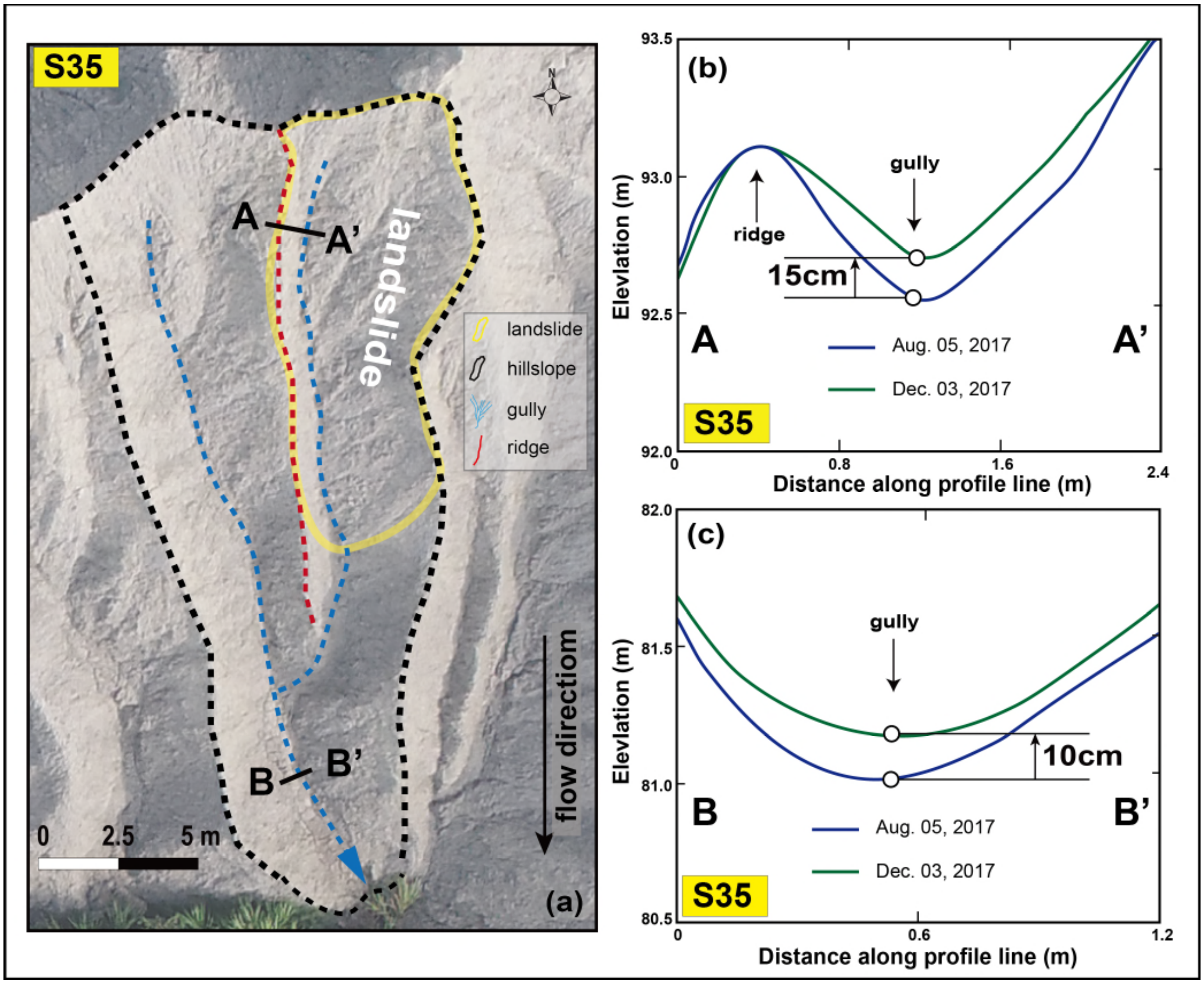

Supplementary Figure 2 Cross-section of the topography in hillslope S35 indicating deposition caused by the landslide. The ortho-images of hillslope were conducted by unmanned aerial vehicle system (UAVs) survey of this study (see Methods). **a** topographic features mapping by ortho-imagery was taken from Dec. 03, 2017. Yellow area denotes landslide, blue and red dashed line denote gully and ridge, respectively. **b** in the cross-section AA' of Supplementary Figure 2-a. The solid lines denote hillslope topography and colors are corresponded to survey date. **c** in the cross-section BB' of Supplementary Figure 2-a. The solid lines denote hillslope topography and colors are corresponded to survey date.

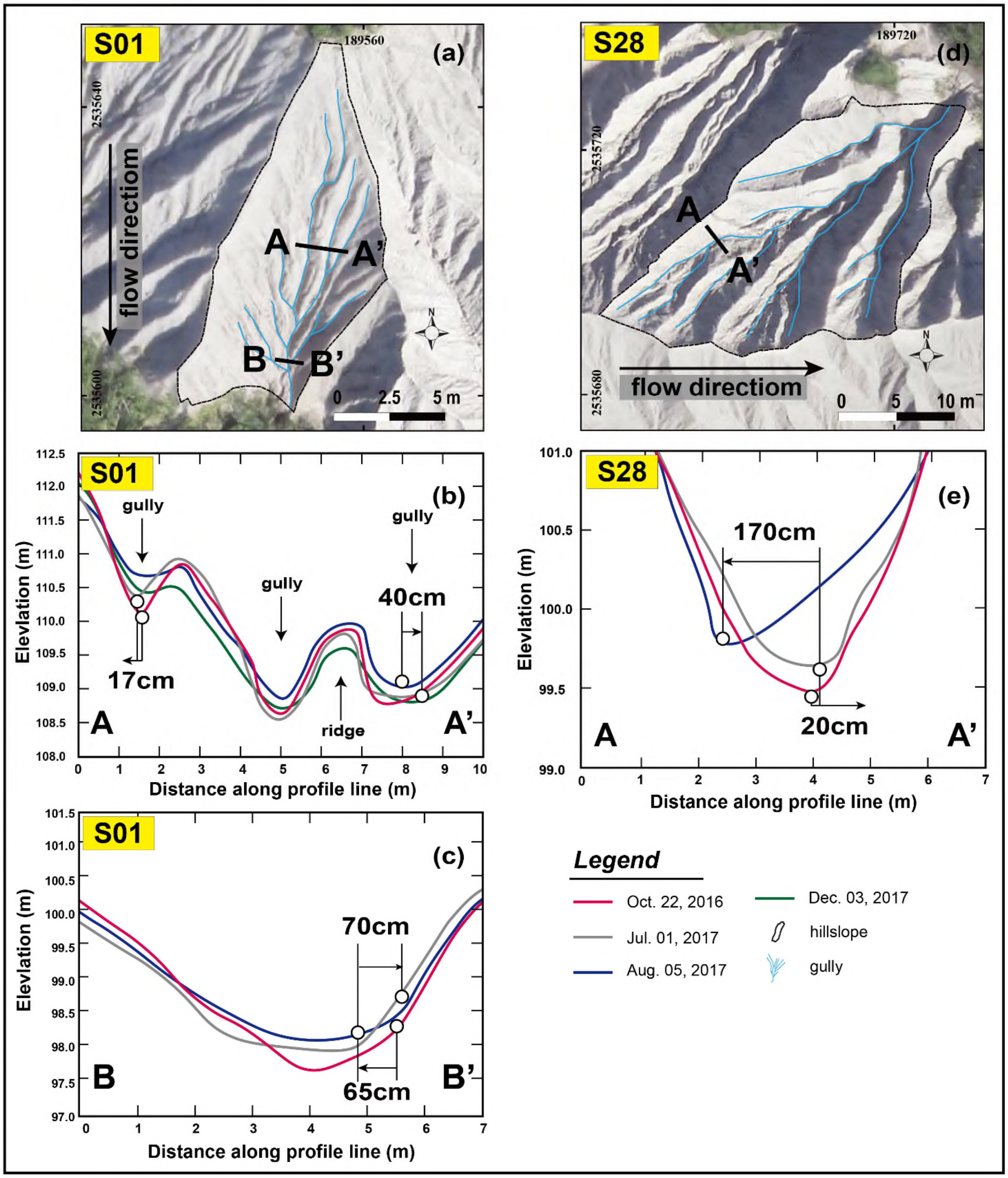

Supplementary Figure 3 Cross-section of the topography in hillslope S01 and S28 indicating gully migration. The ortho-images of hillslope were conducted by UAVs survey of this study (see Methods). **a** ortho-images of hillslope S01 was taken on Oct. 22, 2016. **b** in the cross-section AA' of Supplementary Figure 3-a. The solid lines denote hillslope topography and colors are corresponded to survey date. **c** in the cross-section BB' of Supplementary Figure 3-a. The solid lines denote hillslope topography and colors are corresponded to survey date. **d** ortho-images of hillslope S28 taken on Oct. 22, 2016. **e** in the cross-section AA' of Supplementary Figure 3-d. The solid lines denote hillslope topography and colors are corresponded to survey date.

The following section demonstrate the erosional maps of hillslope in four survey periods.

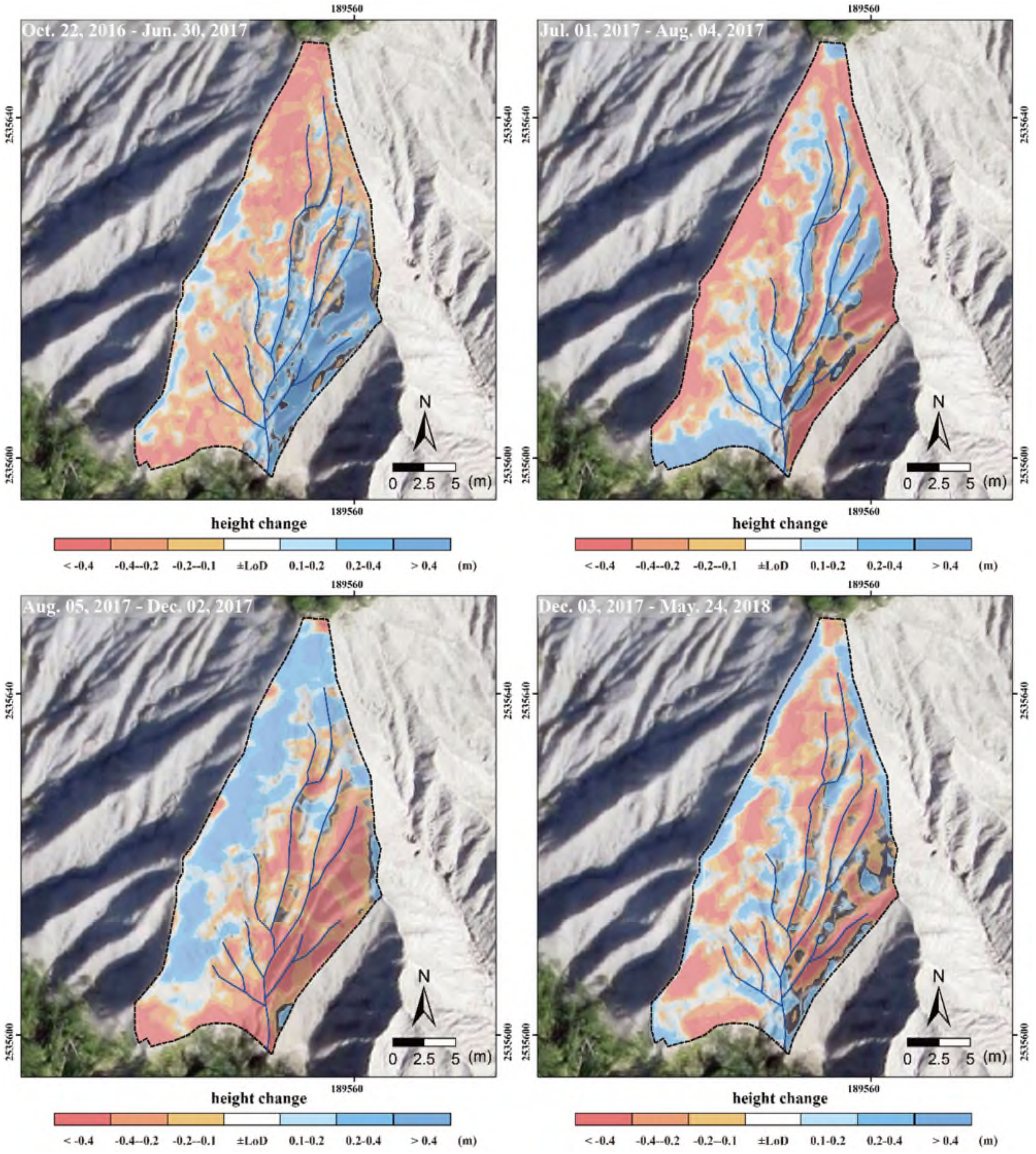

Supplementary Figure 4-1 Distribution of height change obtained from the DEMs by the UAV survey. Blue lines denote drainages. The ortho-images of hillslope were conducted on Oct. 22, 2016 by UAVs survey of this study (see Methods).

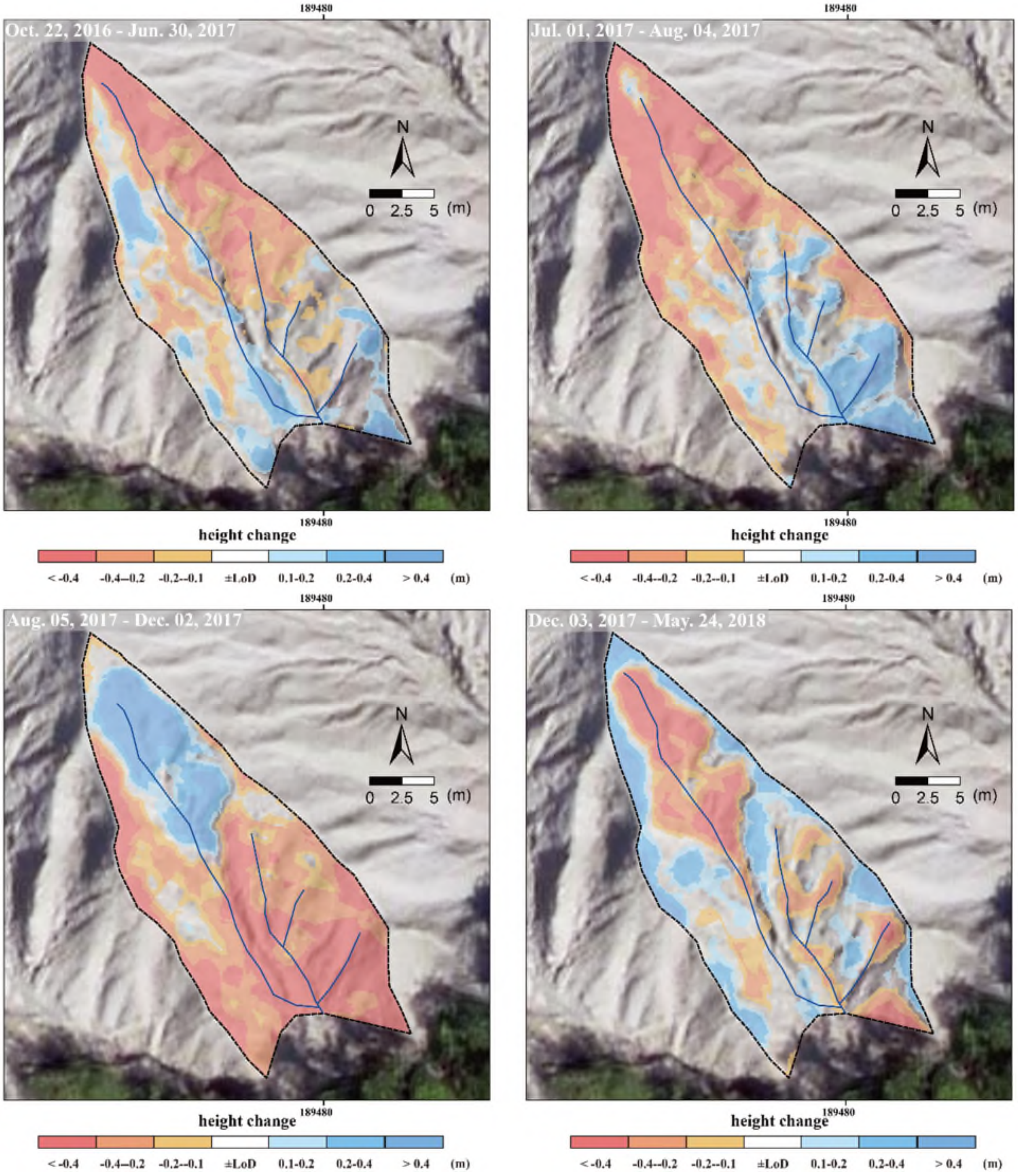

Supplementary Figure 4-2 Distribution of height change obtained from the DEMs by the UAV survey. Blue lines denote drainages. The ortho-images of hillslope were conducted on Oct. 22, 2016 by UAVs survey of this study (see Methods).

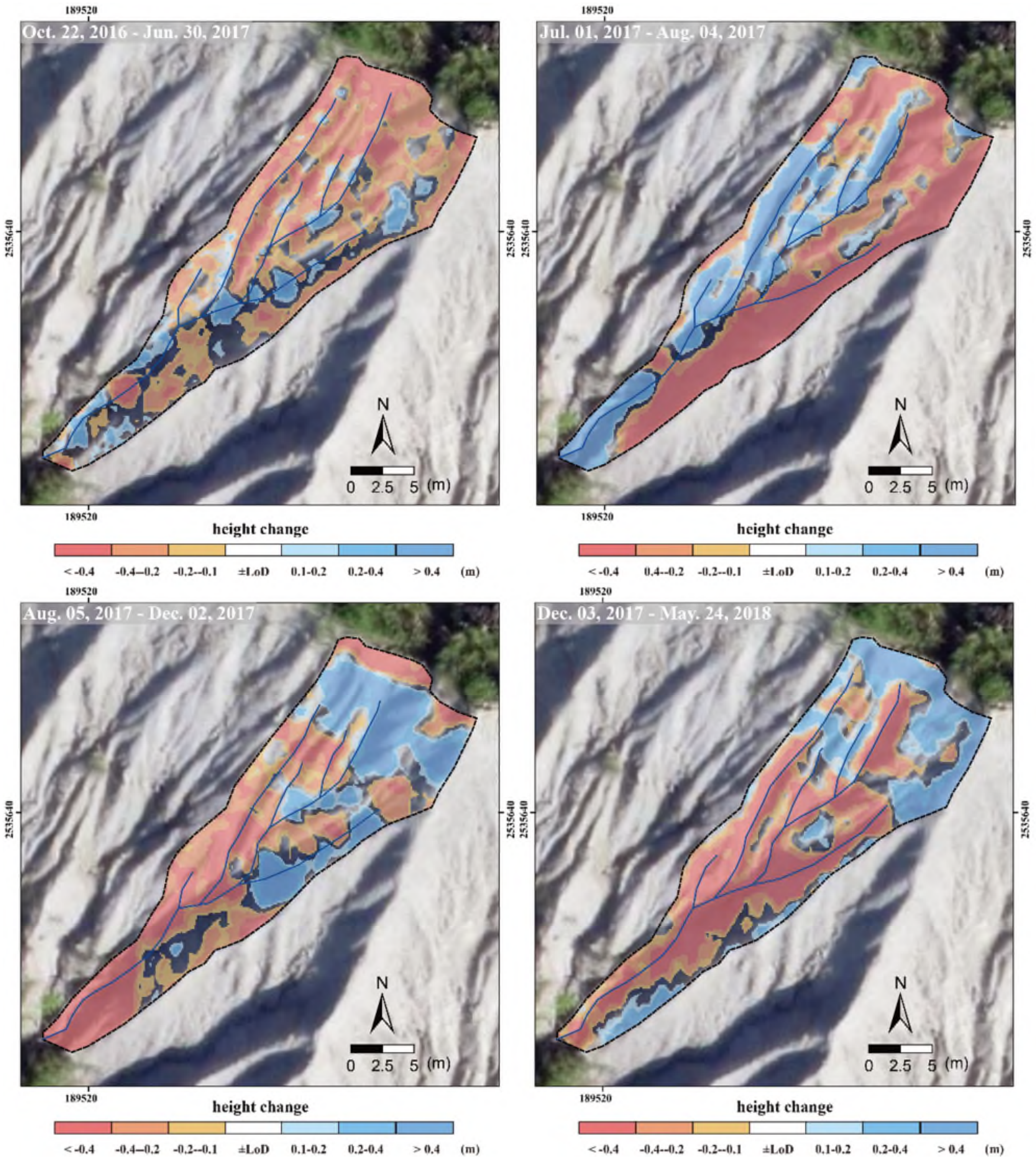

Supplementary Figure 4-3 Distribution of height change obtained from the DEMs by the UAV survey. Blue lines denote drainages. The ortho-images of hillslope were conducted on Oct. 22, 2016 by UAVs survey of this study (see Methods).

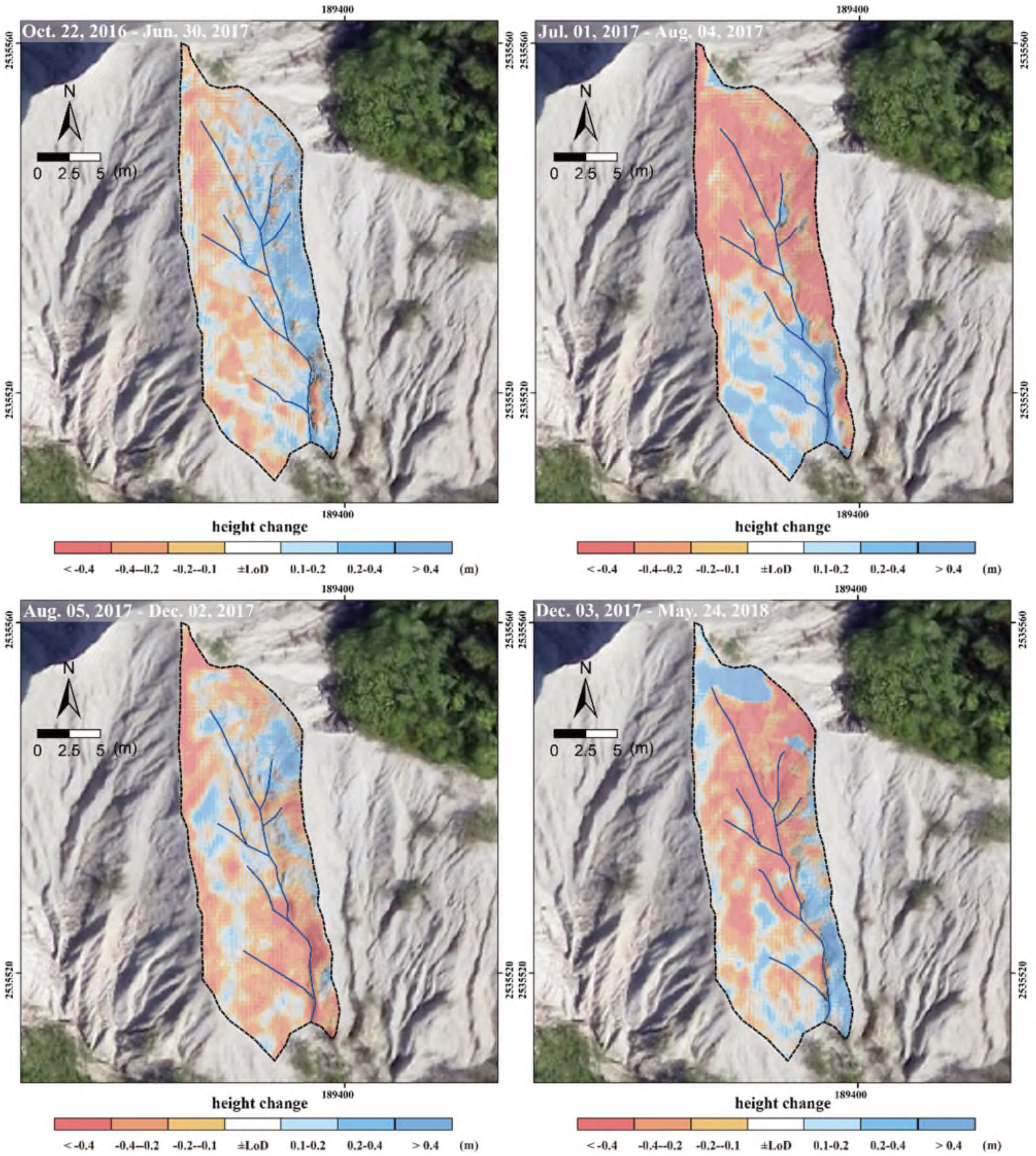

Supplementary Figure 4-4 Distribution of height change obtained from the DEMs by the UAV survey. Blue lines denote drainages. The ortho-images of hillslope were conducted on Oct. 22, 2016 by UAVs survey of this study (see Methods).

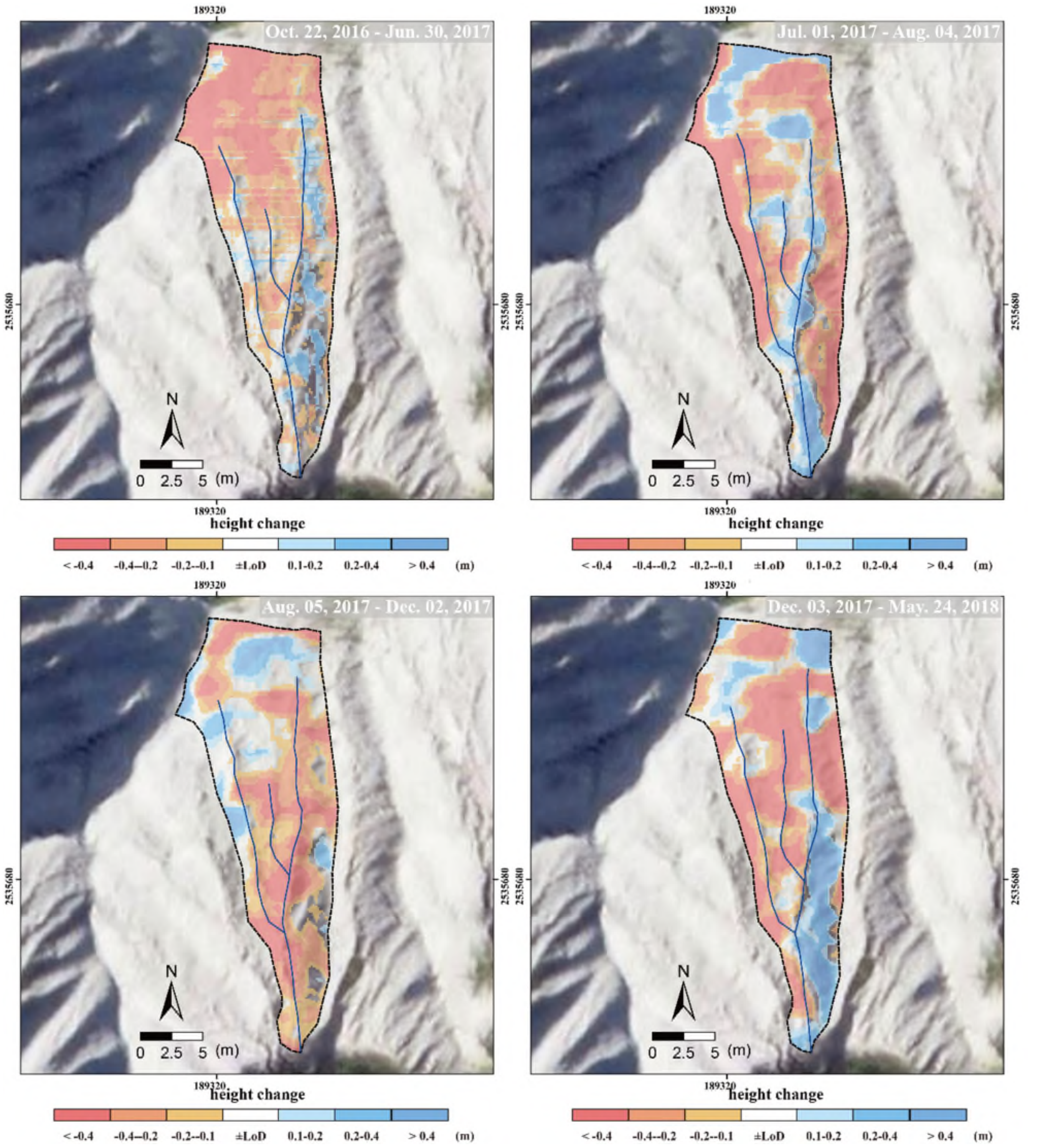

Supplementary Figure 4-5 Distribution of height change obtained from the DEMs by the UAV survey. Blue lines denote drainages. The ortho-images of hillslope were conducted on Oct. 22, 2016 by UAVs survey of this study (see Methods).

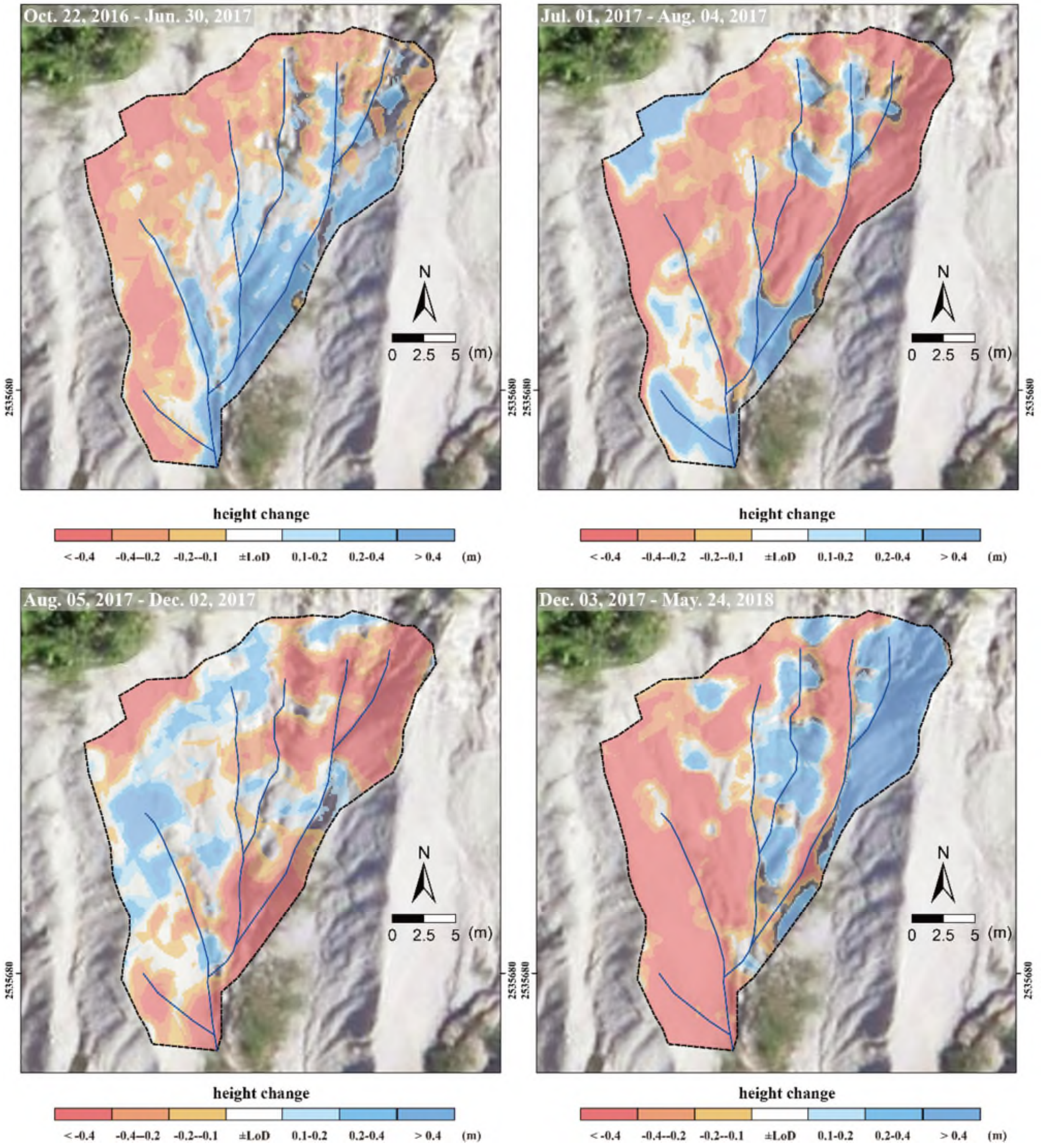

Supplementary Figure 4-6 Distribution of height change obtained from the DEMs by the UAV survey. Blue lines denote drainages. The ortho-images of hillslope were conducted on Oct. 22, 2016 by UAVs survey of this study (see Methods).

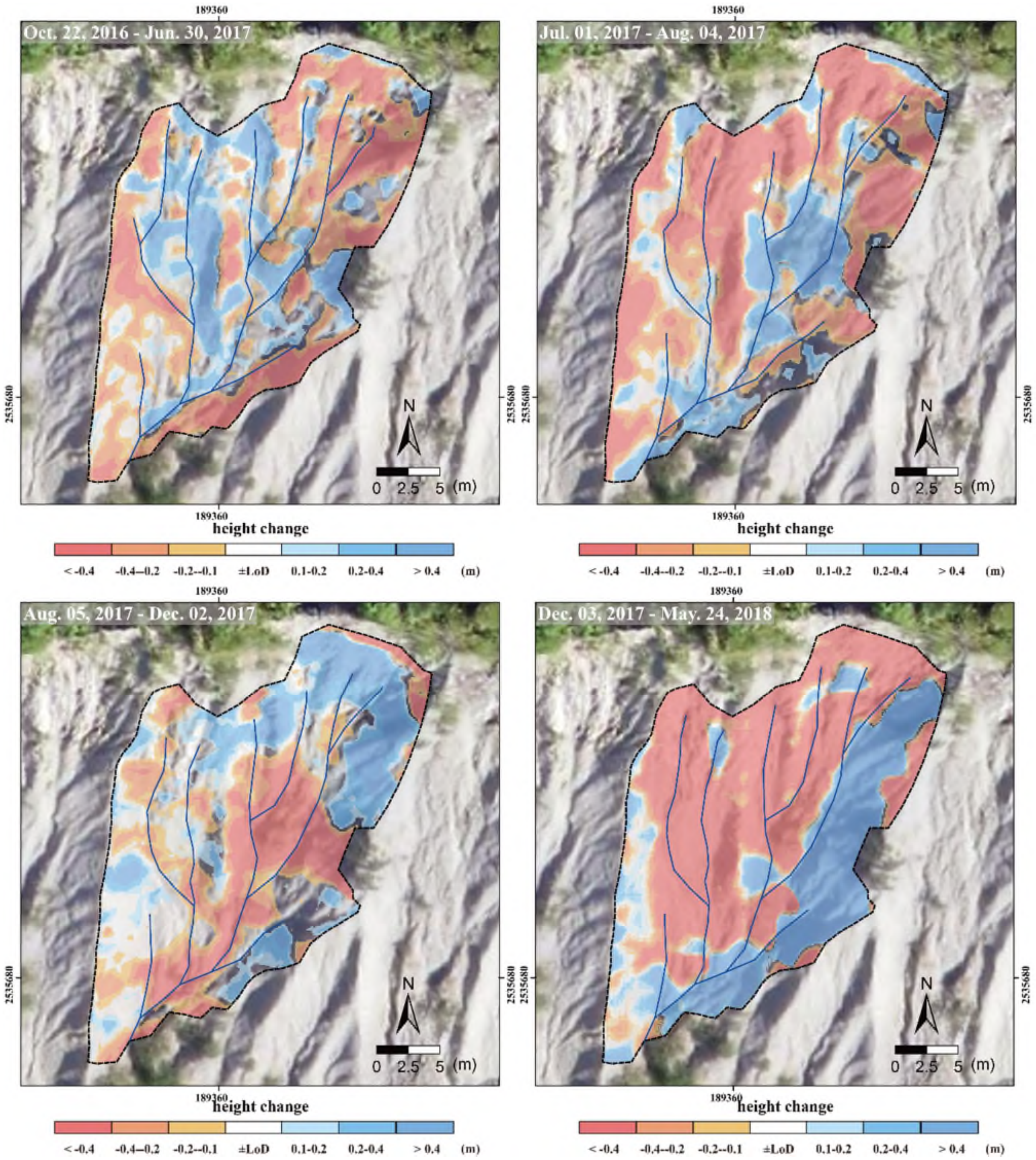

Supplementary Figure 4-7 Distribution of height change obtained from the DEMs by the UAV survey. Blue lines denote drainages. The ortho-images of hillslope were conducted on Oct. 22, 2016 by UAVs survey of this study (see Methods).

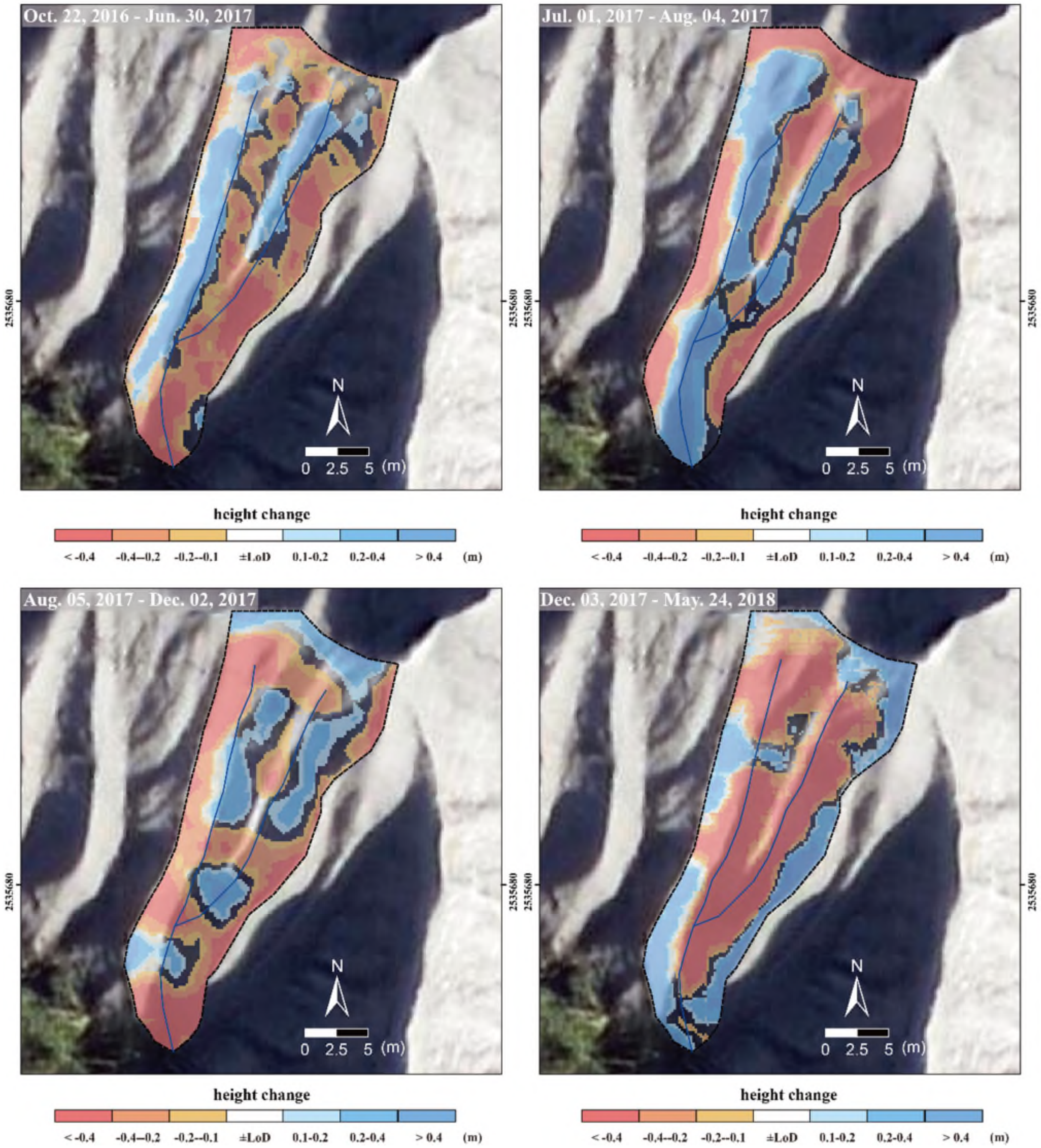

Supplementary Figure 4-8 Distribution of height change obtained from the DEMs by the UAV survey. Blue lines denote drainages. The ortho-images of hillslope were conducted on Oct. 22, 2016 by UAVs survey of this study (see Methods).

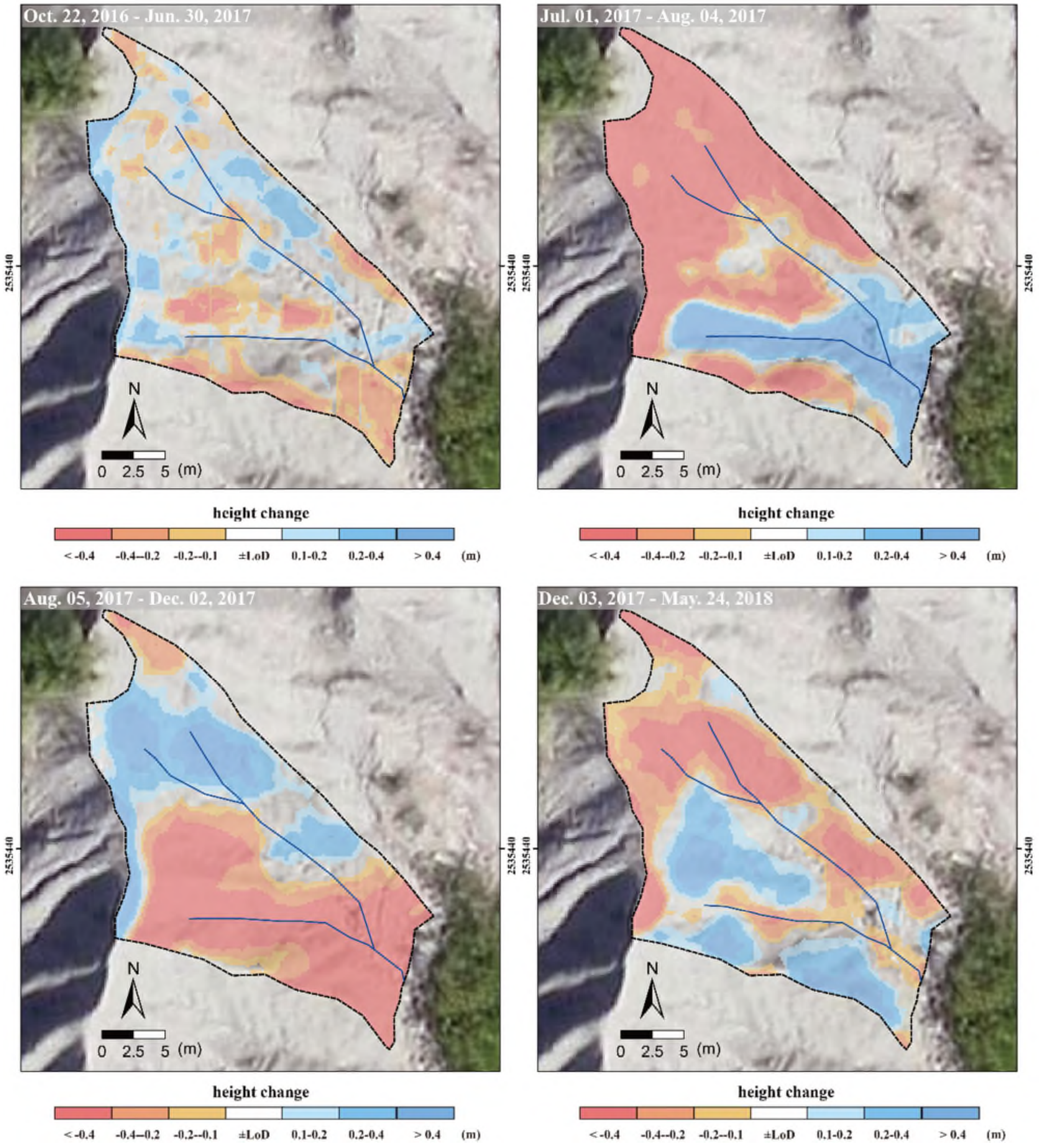

Supplementary Figure 4-9 Distribution of height change obtained from the DEMs by the UAV survey. Blue lines denote drainages. The ortho-images of hillslope were conducted on Oct. 22, 2016 by UAVs survey of this study (see Methods).

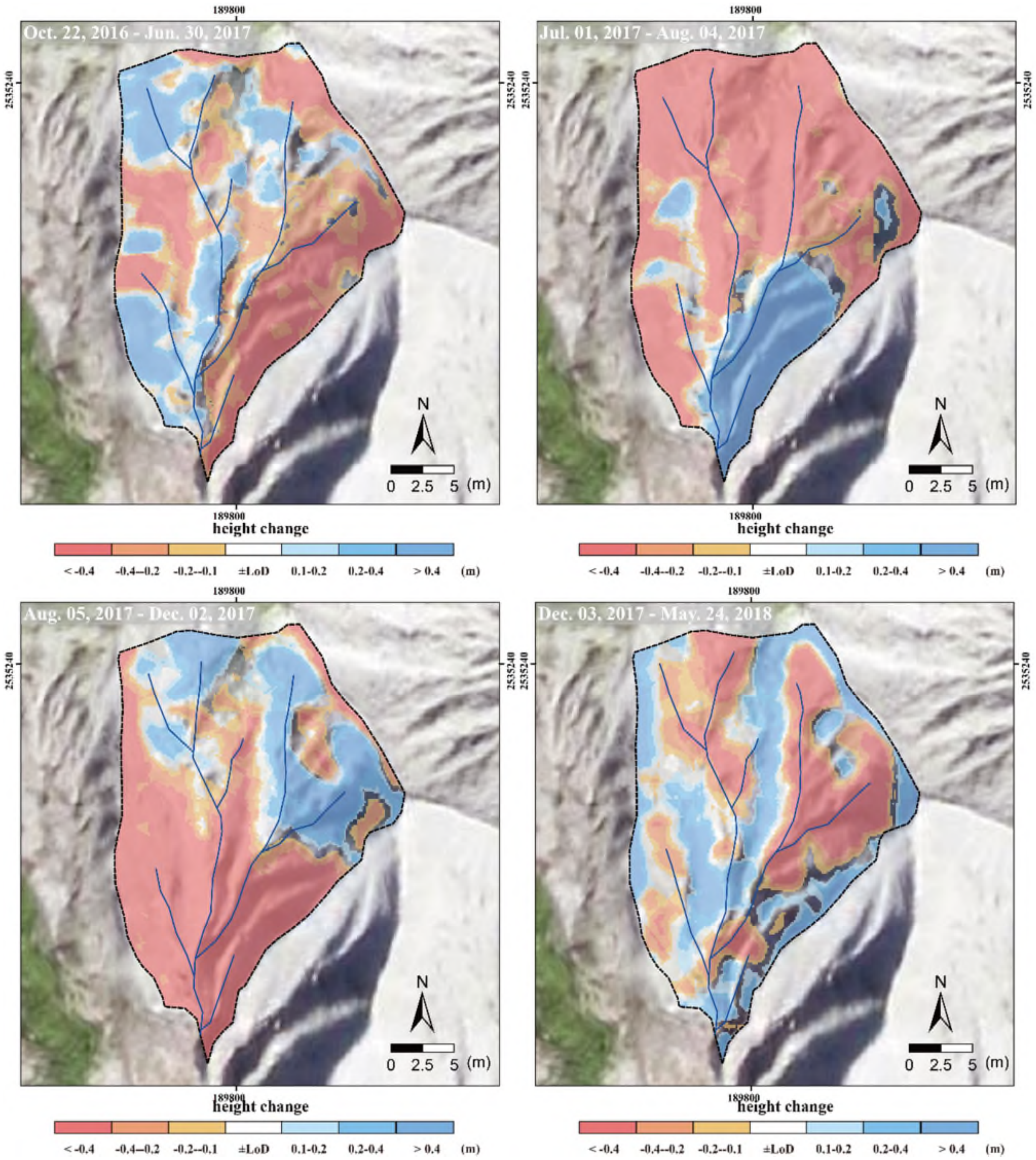

Supplementary Figure 4-10 Distribution of height change obtained from the DEMs by the UAV survey. Blue lines denote drainages. The ortho-images of hillslope were conducted on Oct. 22, 2016 by UAVs survey of this study (see Methods).

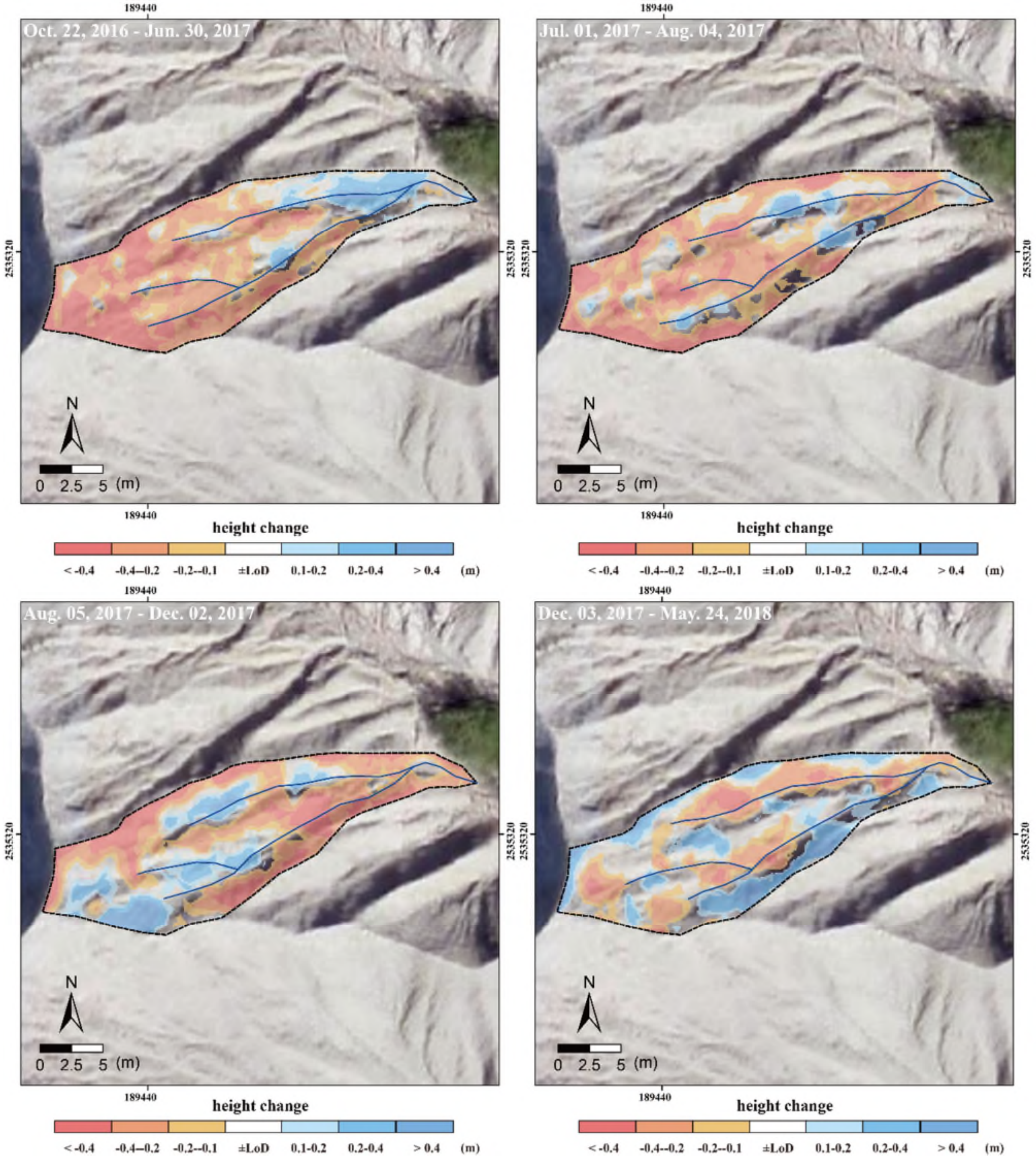

Supplementary Figure 4-11 Distribution of height change obtained from the DEMs by the UAV survey. Blue lines denote drainages. The ortho-images of hillslope were conducted on Oct. 22, 2016 by UAVs survey of this study (see Methods).

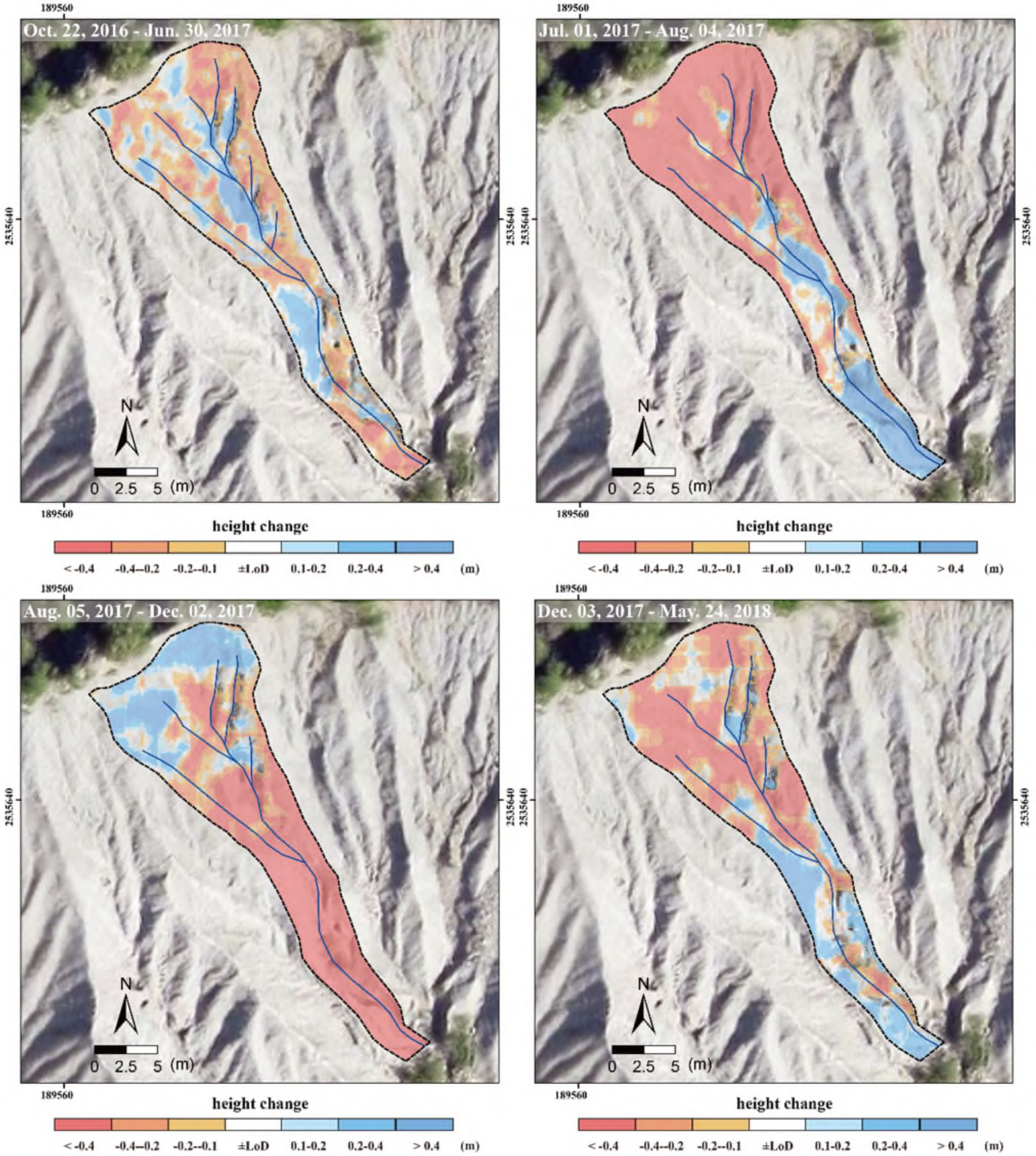

Supplementary Figure 4-12 Distribution of height change obtained from the DEMs by the UAV survey. Blue lines denote drainages. The ortho-images of hillslope were conducted on Oct. 22, 2016 by UAVs survey of this study (see Methods).

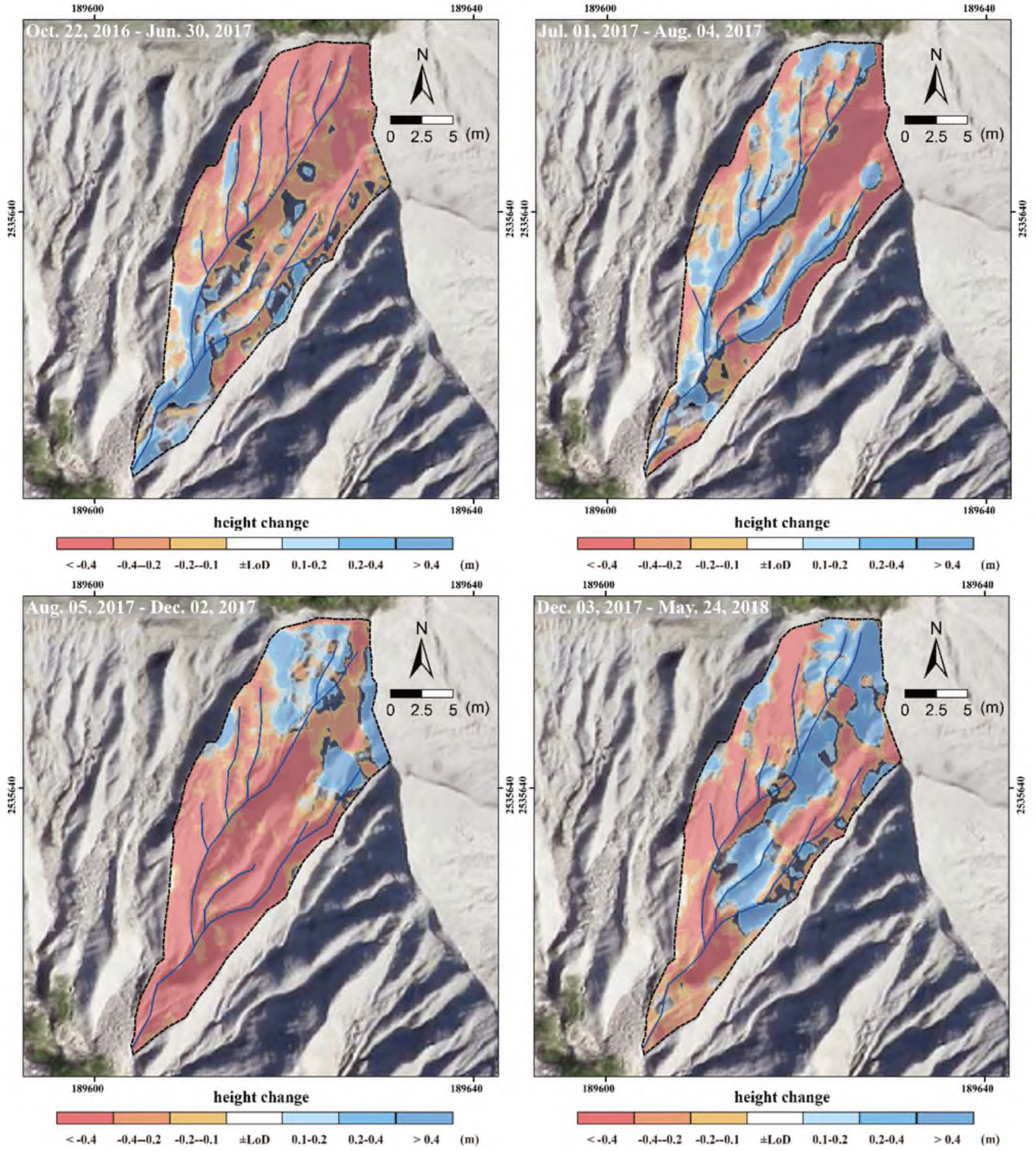

Supplementary Figure 4-13 Distribution of height change obtained from the DEMs by the UAV survey. Blue lines denote drainages. The ortho-images of hillslope were conducted on Oct. 22, 2016 by UAVs survey of this study (see Methods).

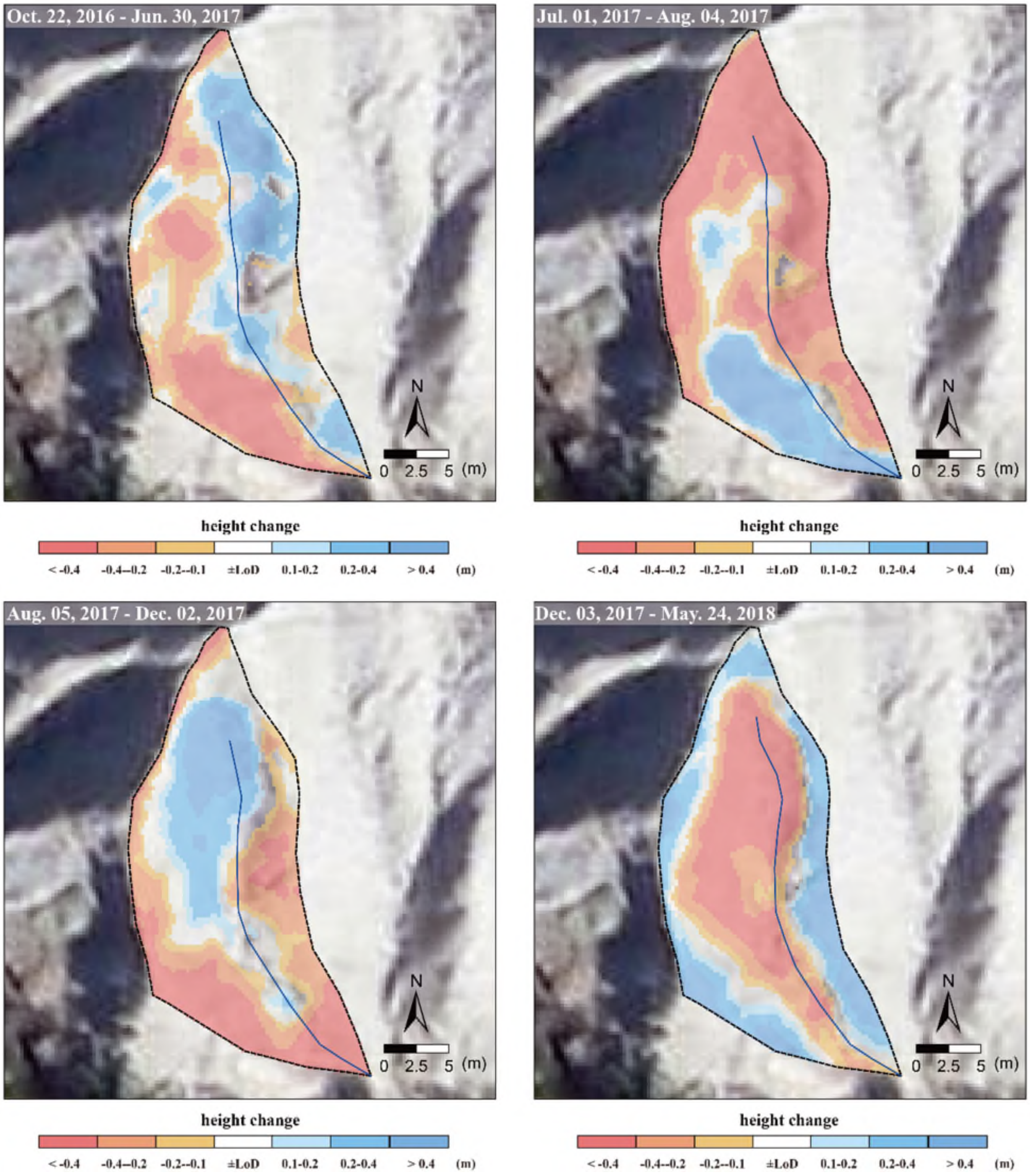

Supplementary Figure 4-14 Distribution of height change obtained from the DEMs by the UAV survey. Blue lines denote drainages. The ortho-images of hillslope were conducted on Oct. 22, 2016 by UAVs survey of this study (see Methods).

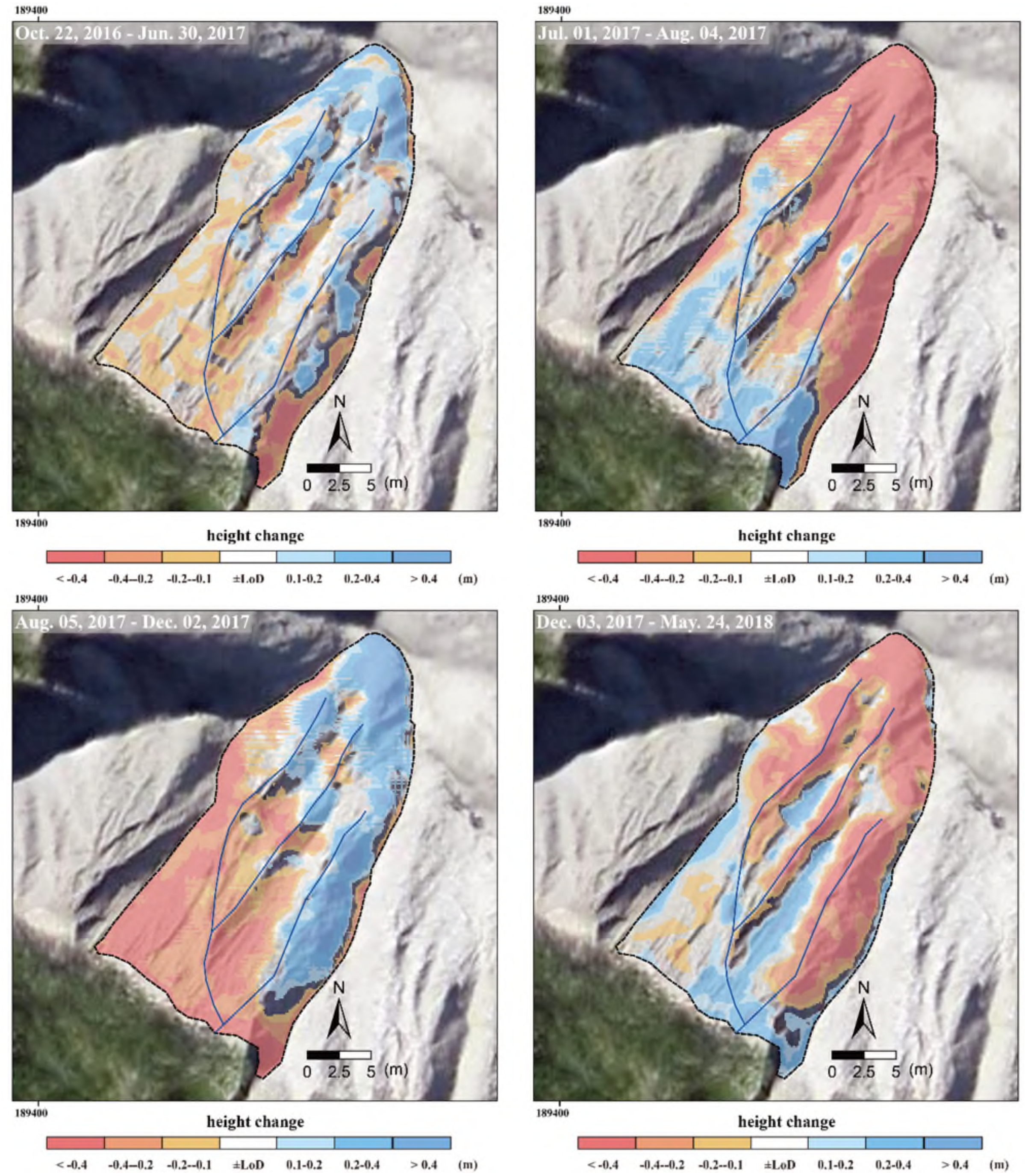

Supplementary Figure 4-15 Distribution of height change obtained from the DEMs by the UAV survey. Blue lines denote drainages. The ortho-images of hillslope were conducted on Oct. 22, 2016 by UAVs survey of this study (see Methods).

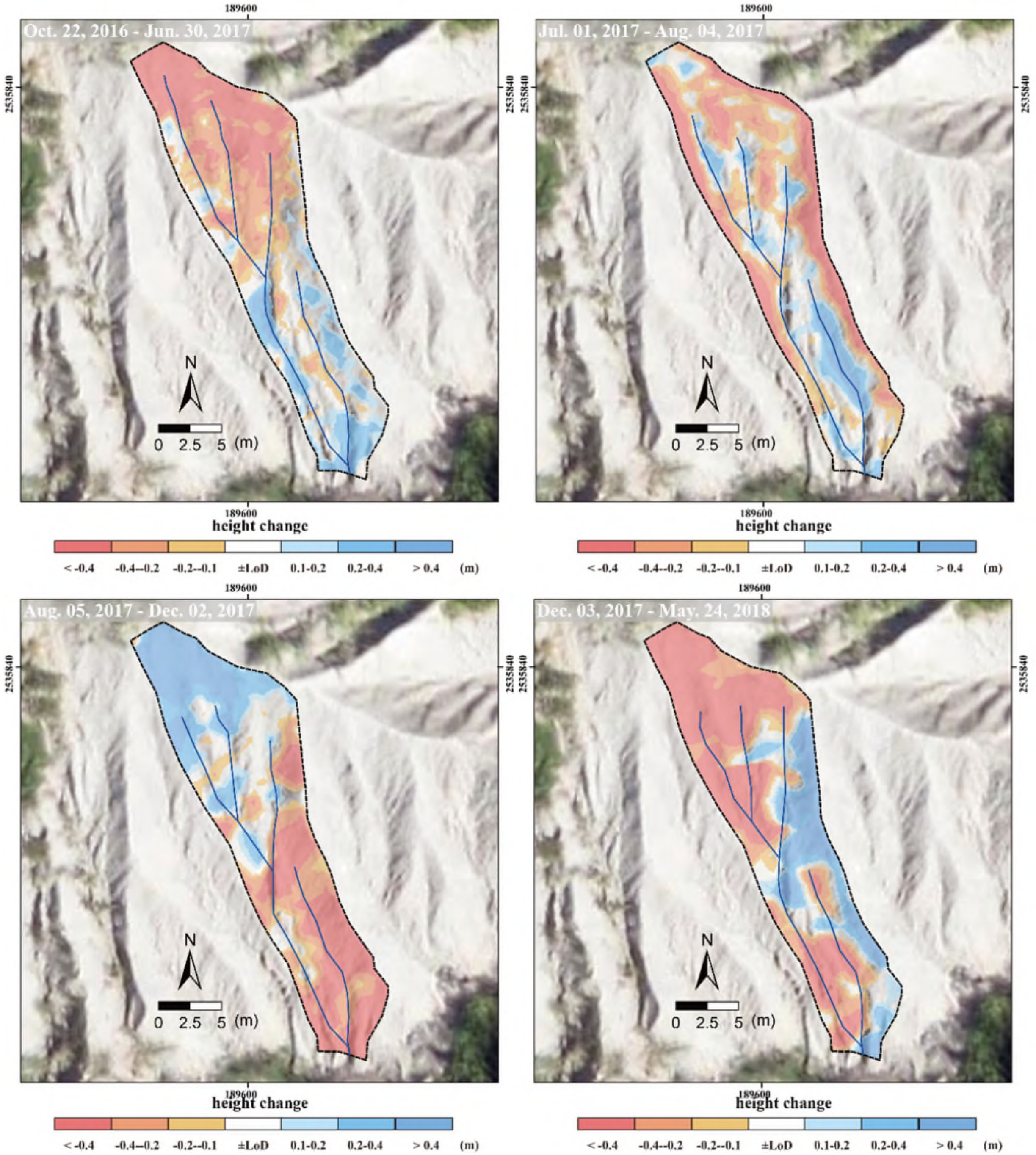

Supplementary Figure 4-16 Distribution of height change obtained from the DEMs by the UAV survey. Blue lines denote drainages. The ortho-images of hillslope were conducted on Oct. 22, 2016 by UAVs survey of this study (see Methods).

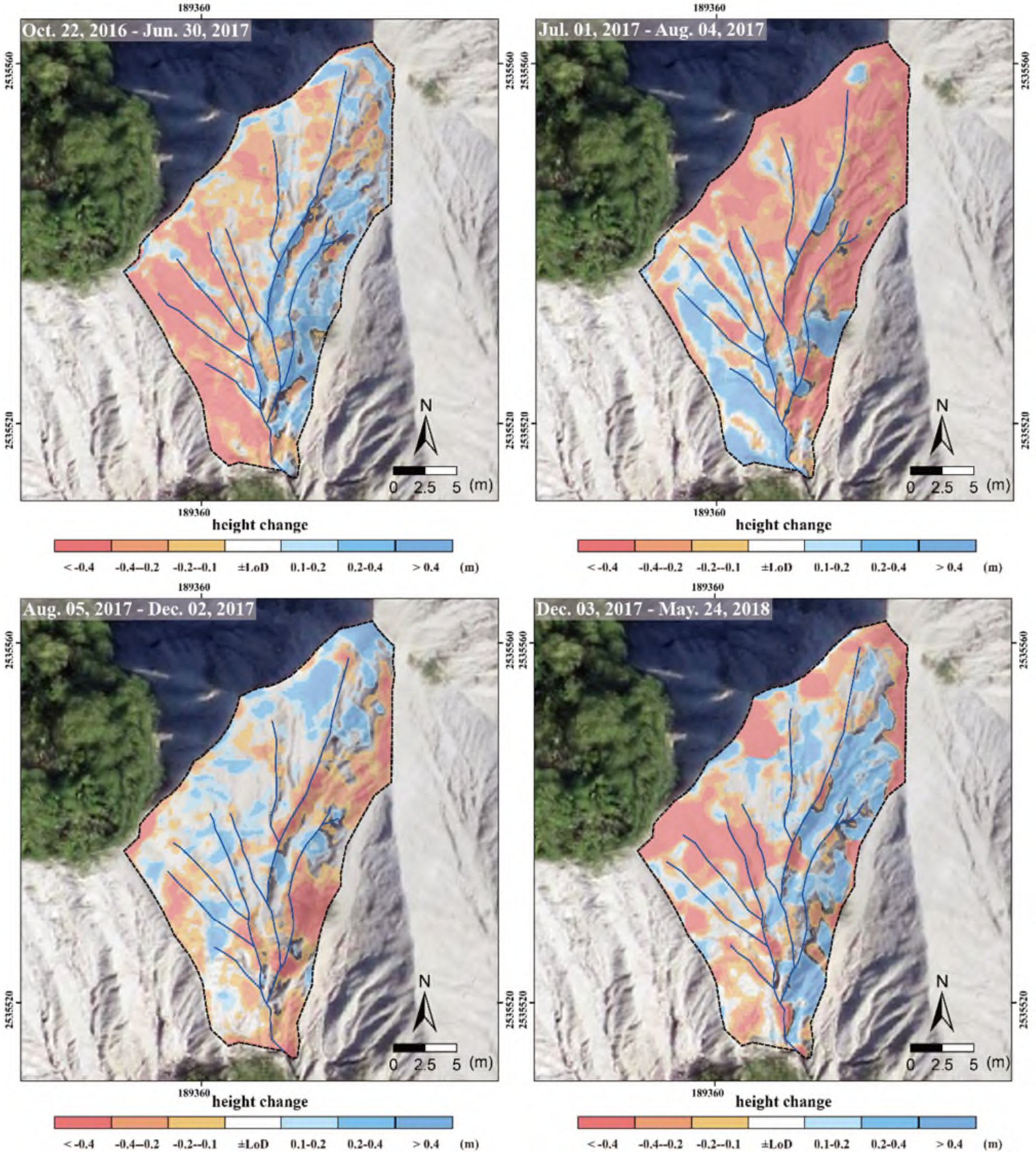

Supplementary Figure 4-17 Distribution of height change obtained from the DEMs by the UAV survey. Blue lines denote drainages. The ortho-images of hillslope were conducted on Oct. 22, 2016 by UAVs survey of this study (see Methods).

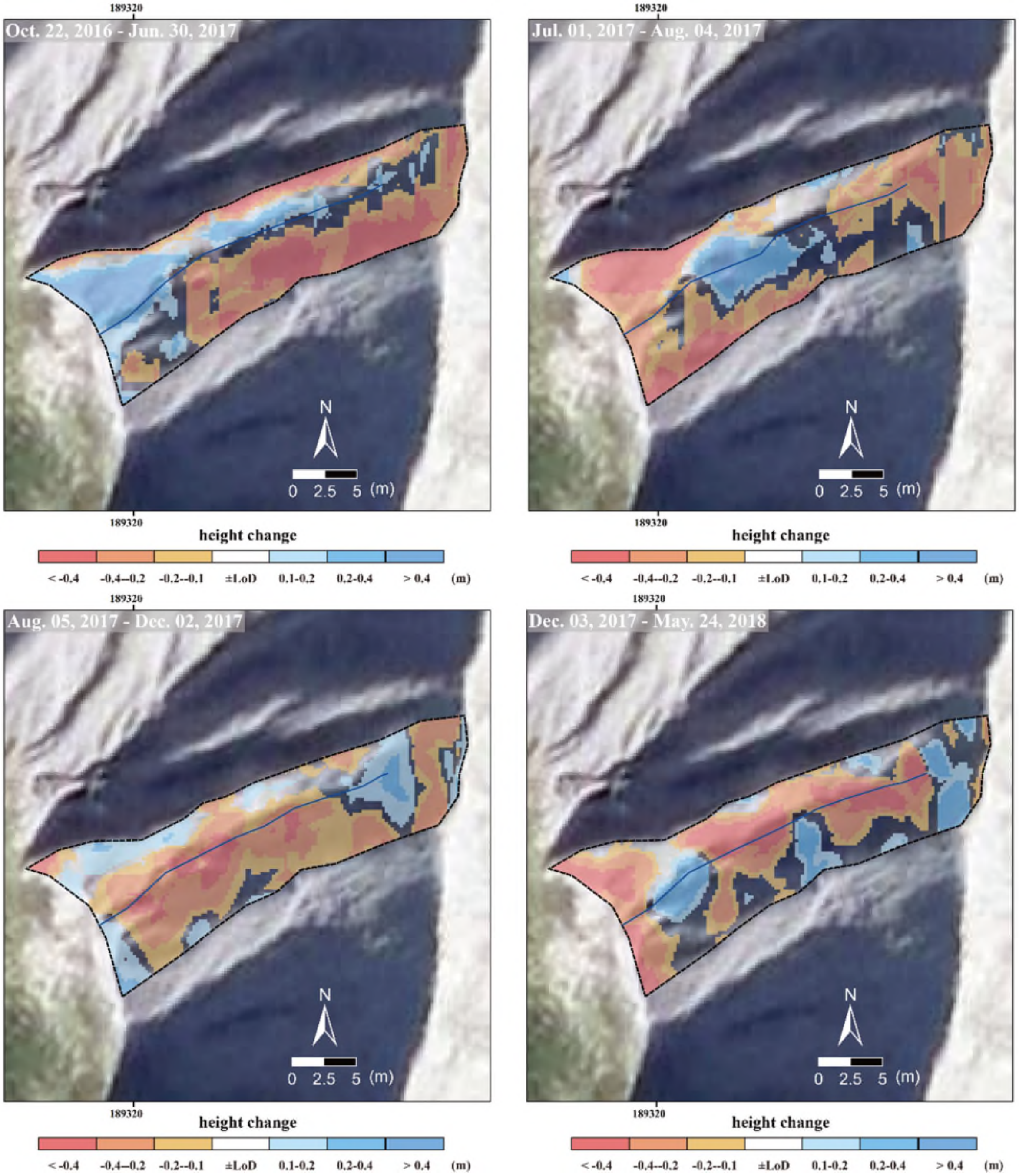

Supplementary Figure 4-18 Distribution of height change obtained from the DEMs by the UAV survey. Blue lines denote drainages. The ortho-images of hillslope were conducted on Oct. 22, 2016 by UAVs survey of this study (see Methods).

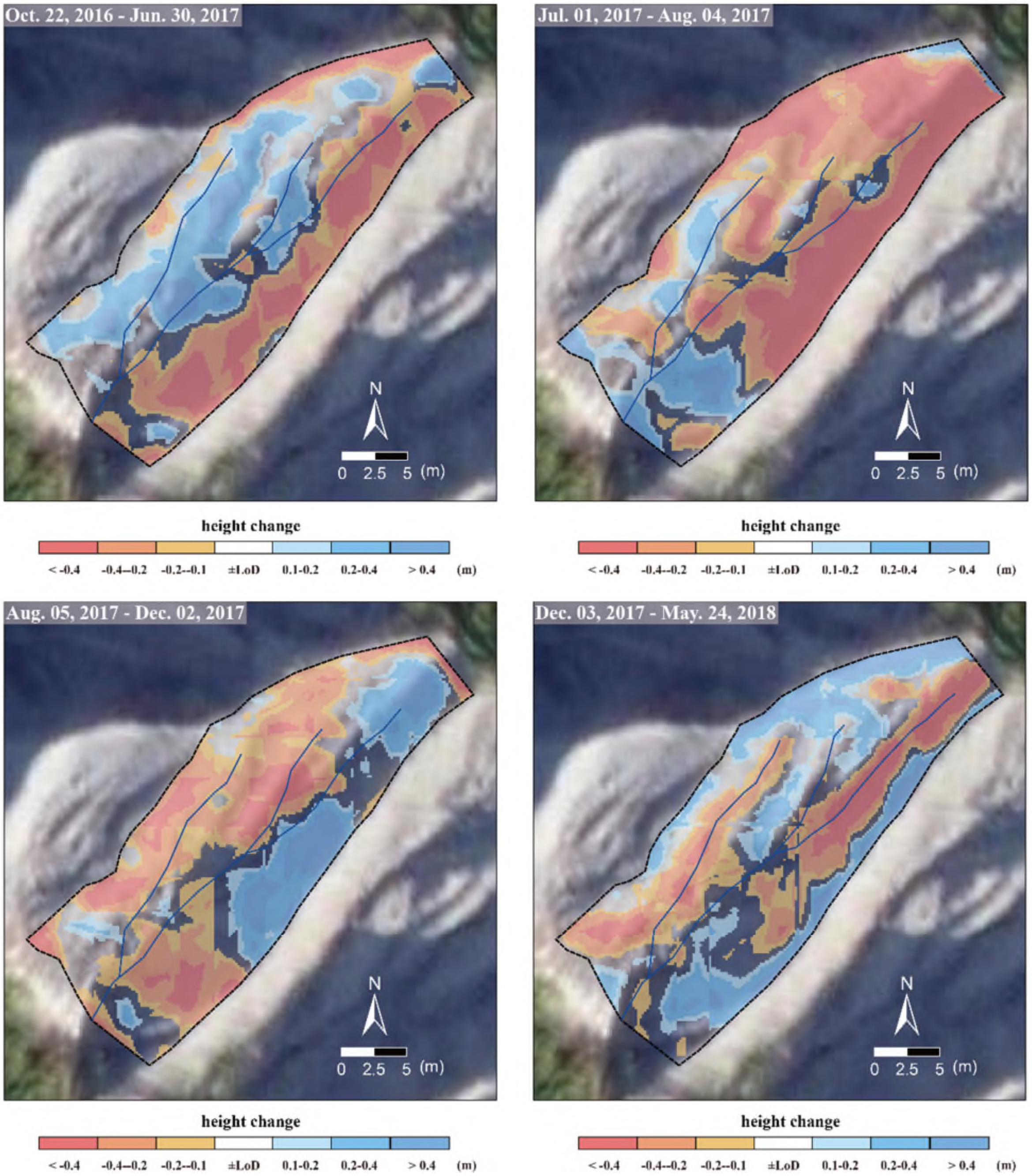

Supplementary Figure 4-19 Distribution of height change obtained from the DEMs by the UAV survey. Blue lines denote drainages. The ortho-images of hillslope were conducted on Oct. 22, 2016 by UAVs survey of this study (see Methods).

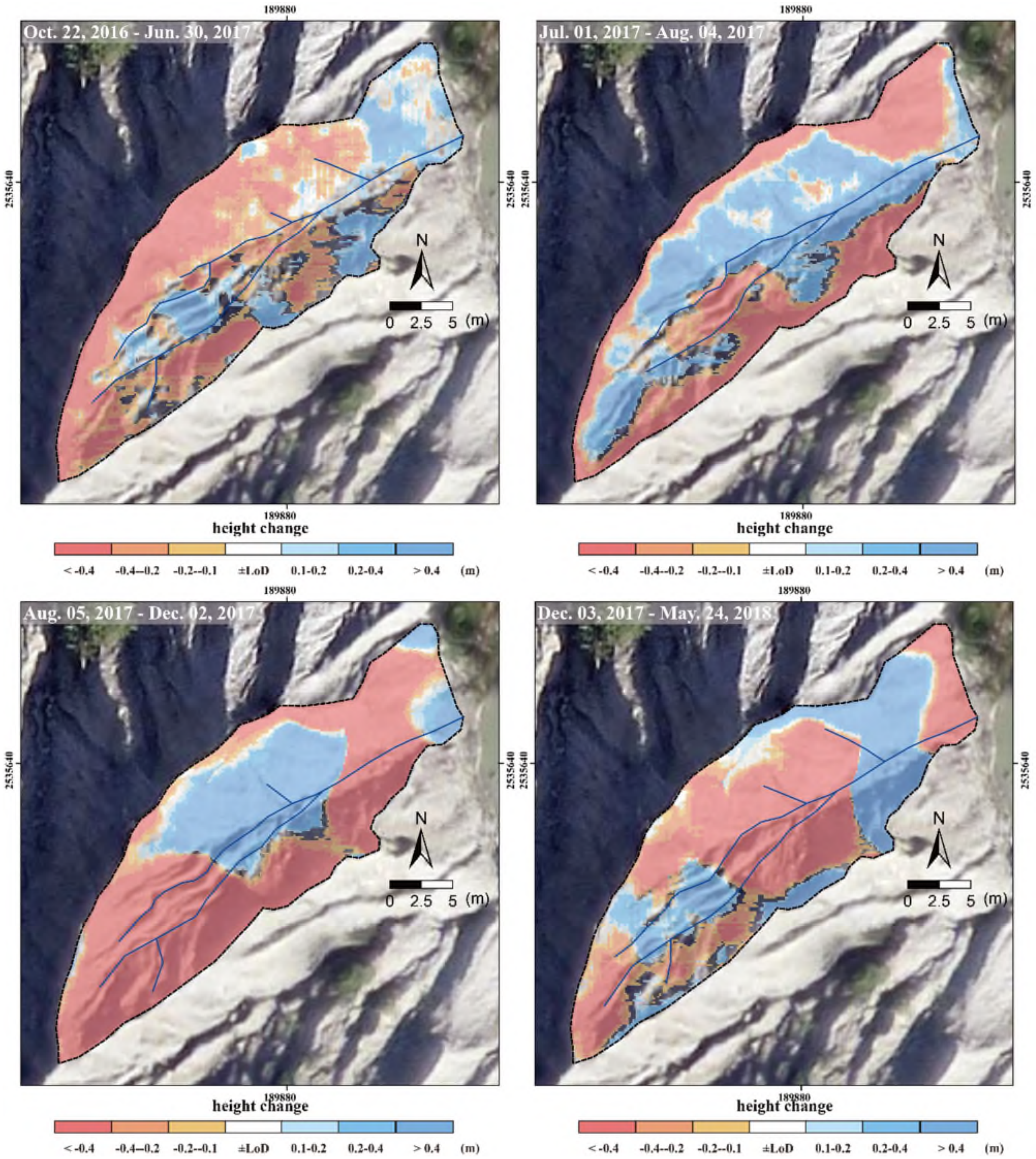

Supplementary Figure 4-20 Distribution of height change obtained from the DEMs by the UAV survey. Blue lines denote drainages. The ortho-images of hillslope were conducted on Oct. 22, 2016 by UAVs survey of this study (see Methods).

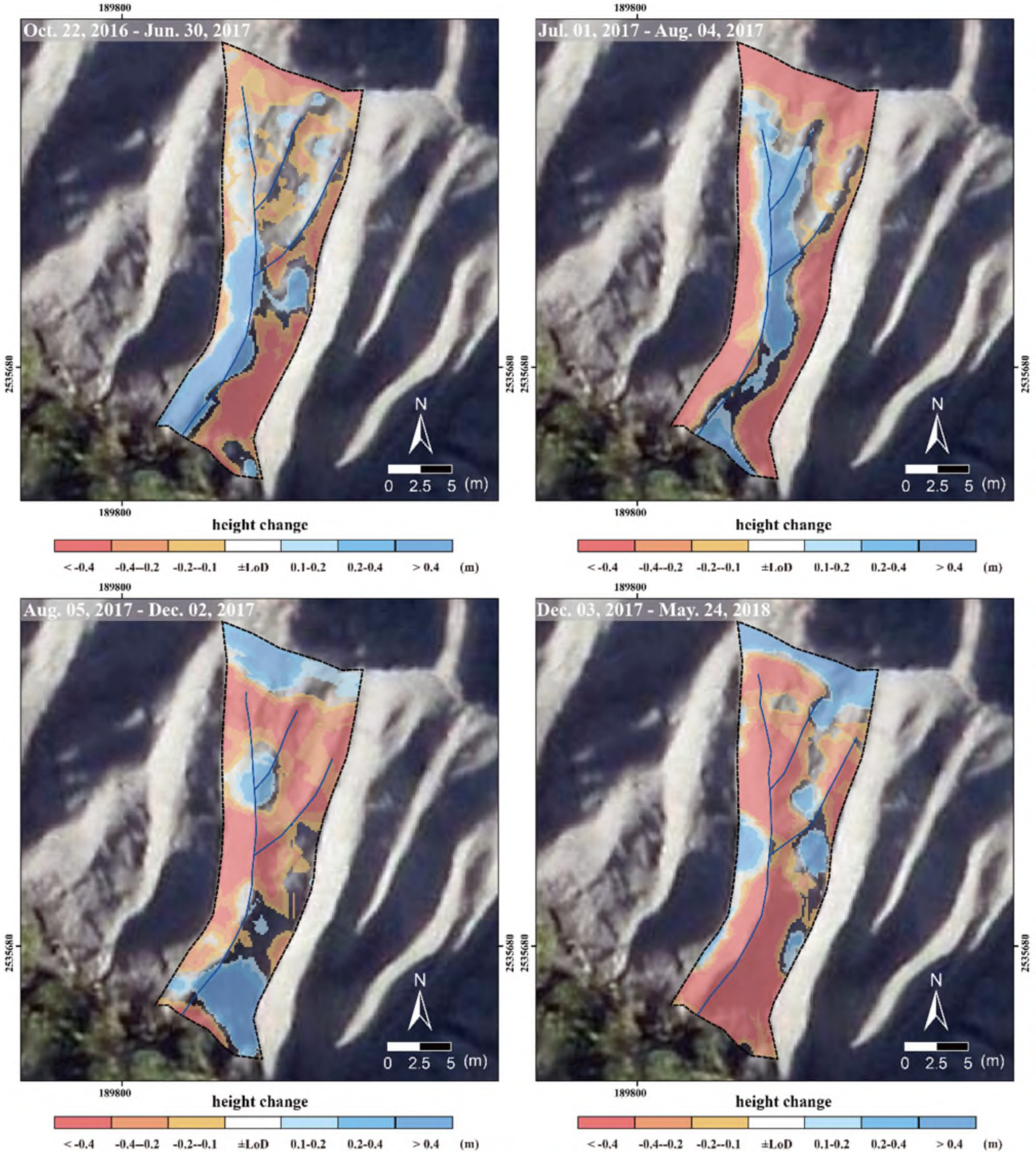

Supplementary Figure 4-21 Distribution of height change obtained from the DEMs by the UAV survey. Blue lines denote drainages. The ortho-images of hillslope were conducted on Oct. 22, 2016 by UAVs survey of this study (see Methods).

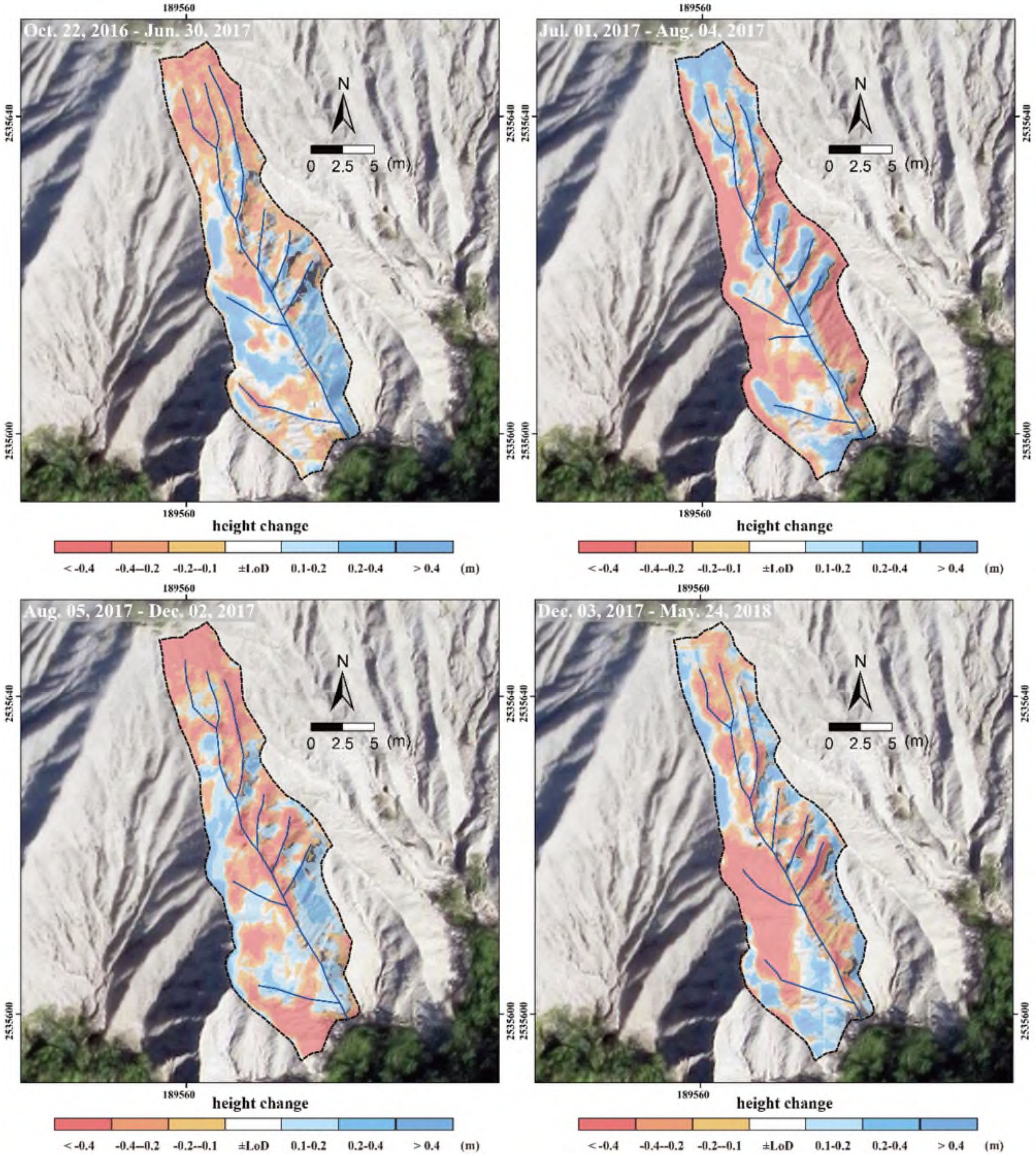

Supplementary Figure 4-22 Distribution of height change obtained from the DEMs by the UAV survey. Blue lines denote drainages. The ortho-images of hillslope were conducted on Oct. 22, 2016 by UAVs survey of this study (see Methods).

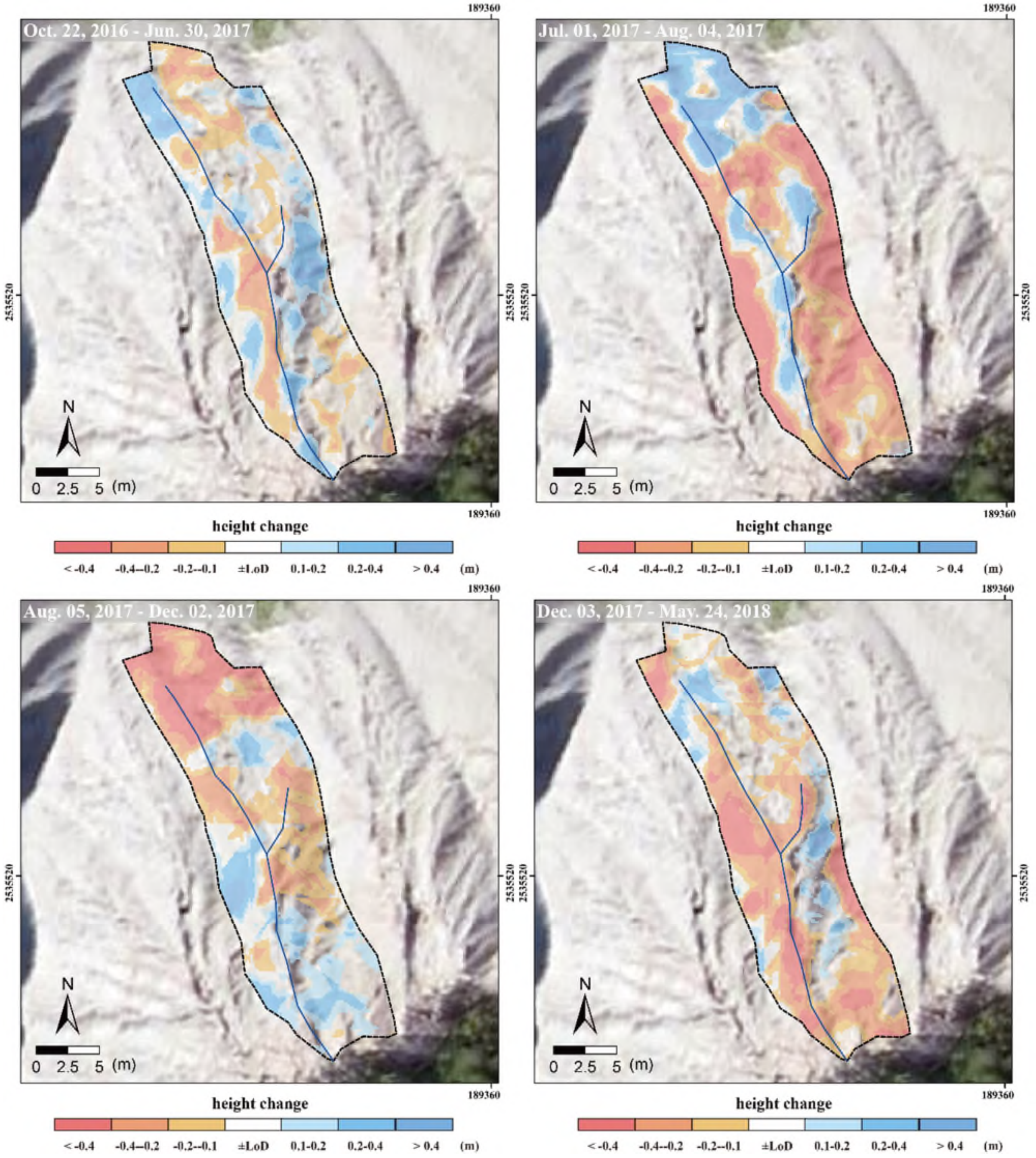

Supplementary Figure 4-23 Distribution of height change obtained from the DEMs by the UAV survey. Blue lines denote drainages. The ortho-images of hillslope were conducted on Oct. 22, 2016 by UAVs survey of this study (see Methods).

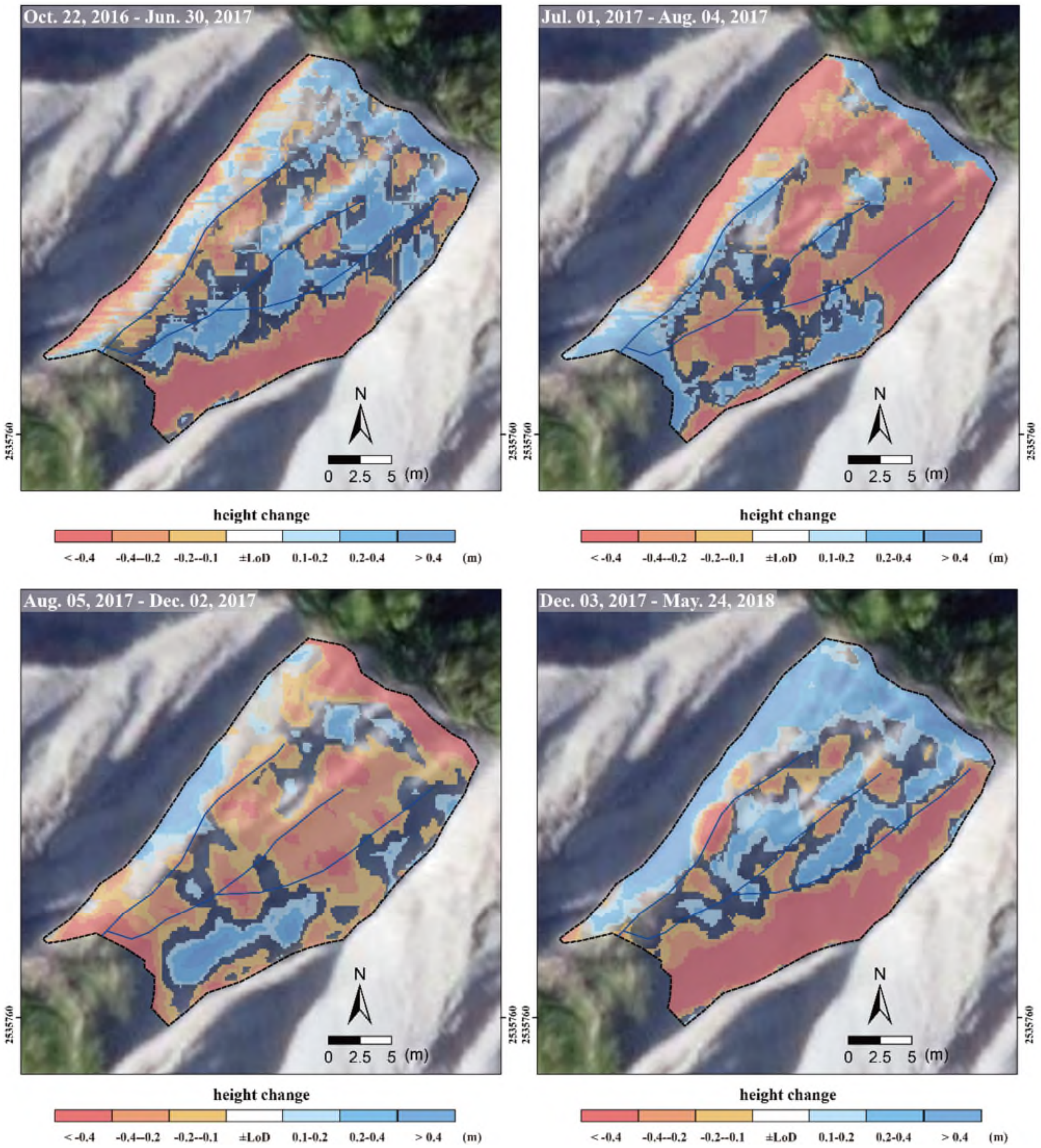

Supplementary Figure 4-24 Distribution of height change obtained from the DEMs by the UAV survey. Blue lines denote drainages. The ortho-images of hillslope were conducted on Oct. 22, 2016 by UAVs survey of this study (see Methods).

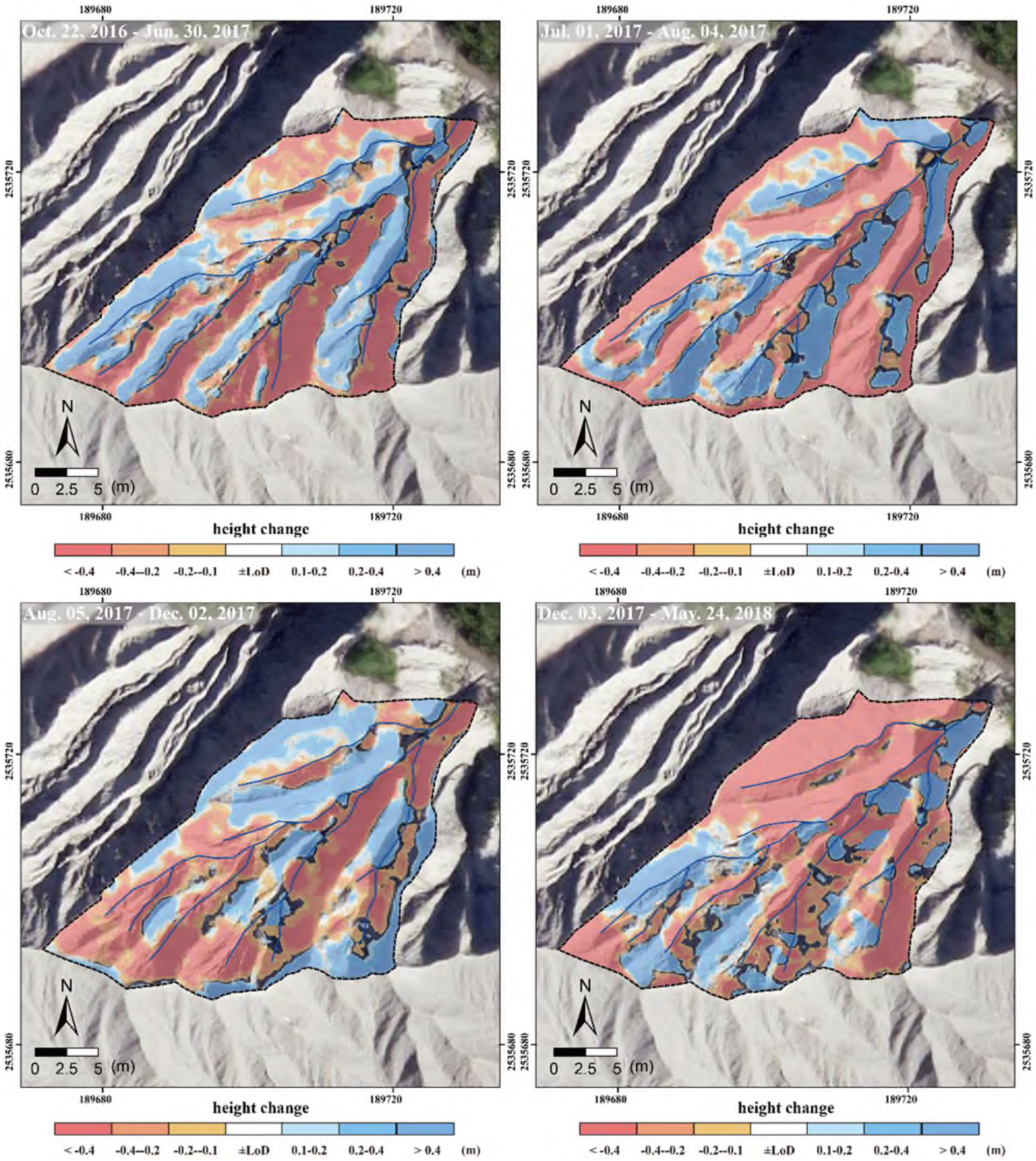

Supplementary Figure 4-25 Distribution of height change obtained from the DEMs by the UAV survey. Blue lines denote drainages. The ortho-images of hillslope were conducted on Oct. 22, 2016 by UAVs survey of this study (see Methods).

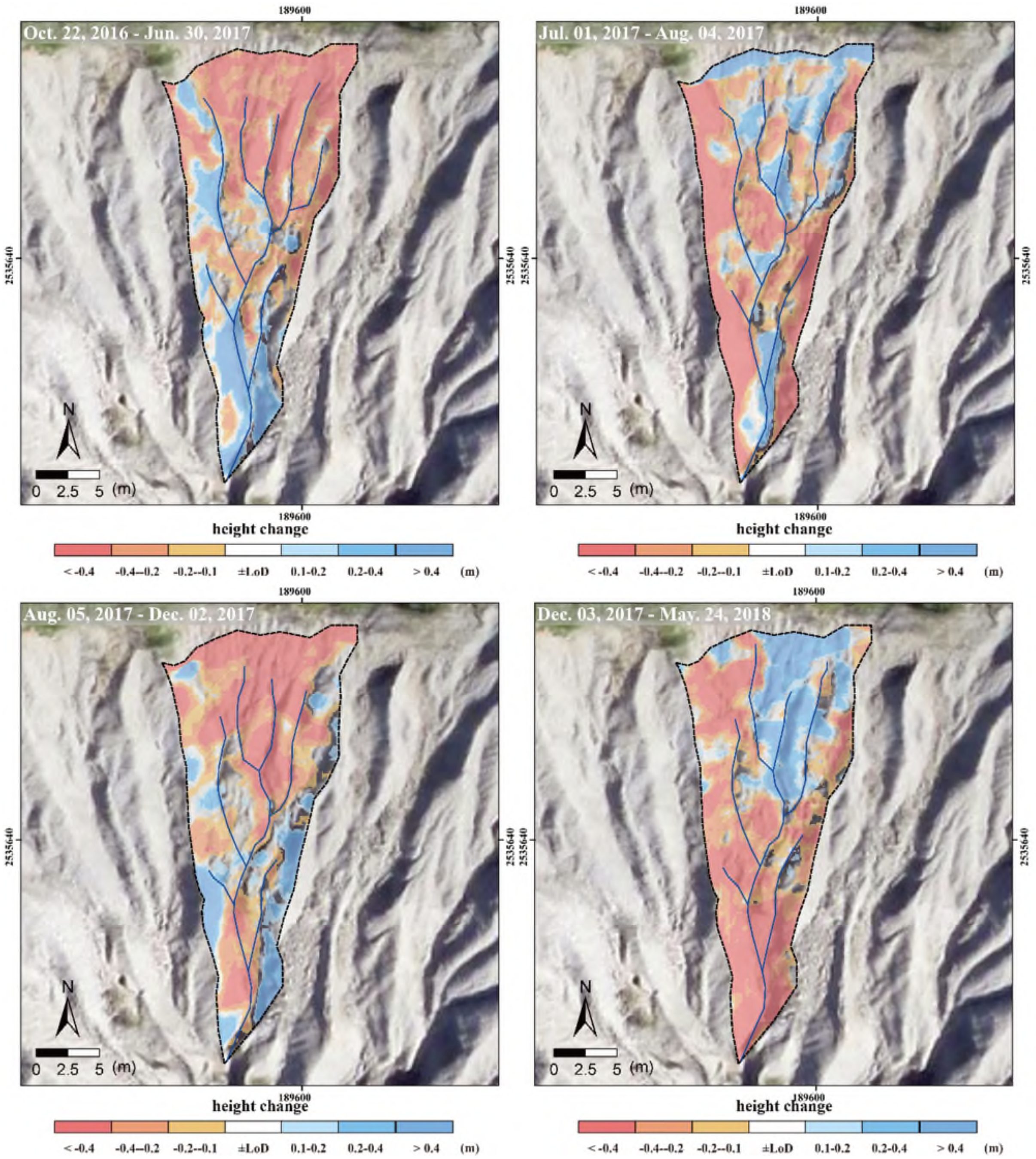

Supplementary Figure 4-26 Distribution of height change obtained from the DEMs by the UAV survey. Blue lines denote drainages. The ortho-images of hillslope were conducted on Oct. 22, 2016 by UAVs survey of this study (see Methods).

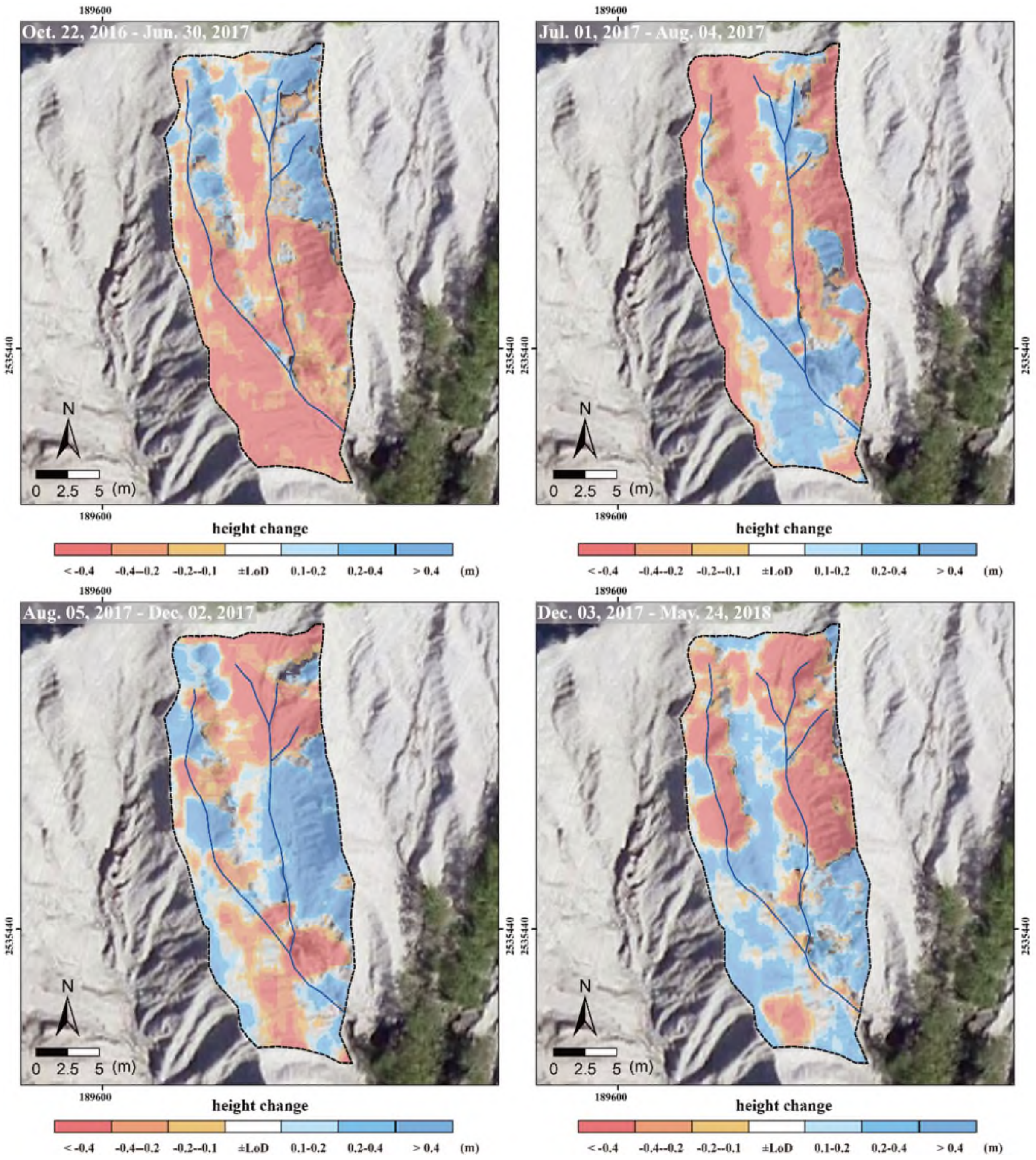

Supplementary Figure 4-27 Distribution of height change obtained from the DEMs by the UAV survey. Blue lines denote drainages. The ortho-images of hillslope were conducted on Oct. 22, 2016 by UAVs survey of this study (see Methods).

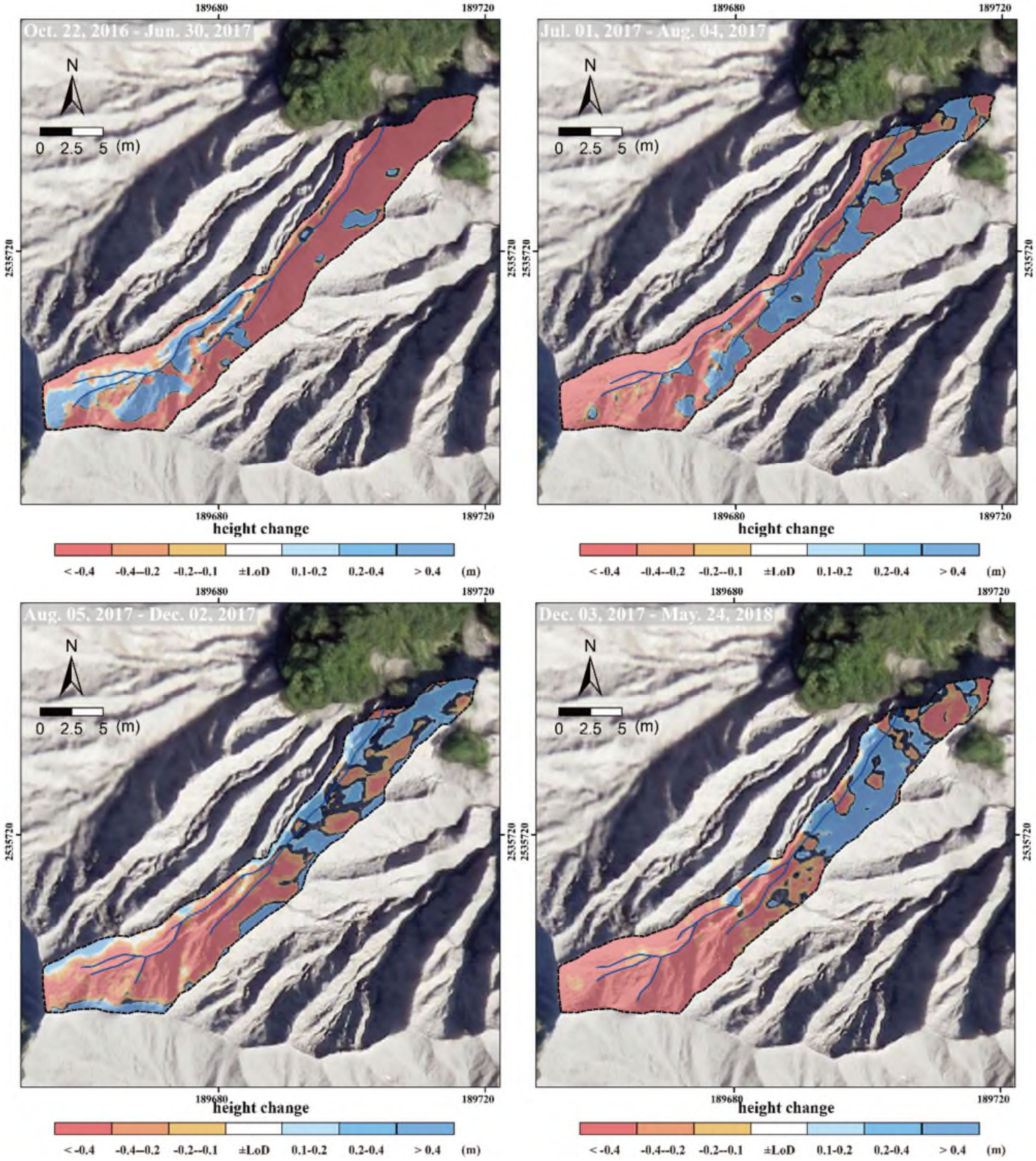

Supplementary Figure 4-28 Distribution of height change obtained from the DEMs by the UAV survey. Blue lines denote drainages. The ortho-images of hillslope were conducted on Oct. 22, 2016 by UAVs survey of this study (see Methods).

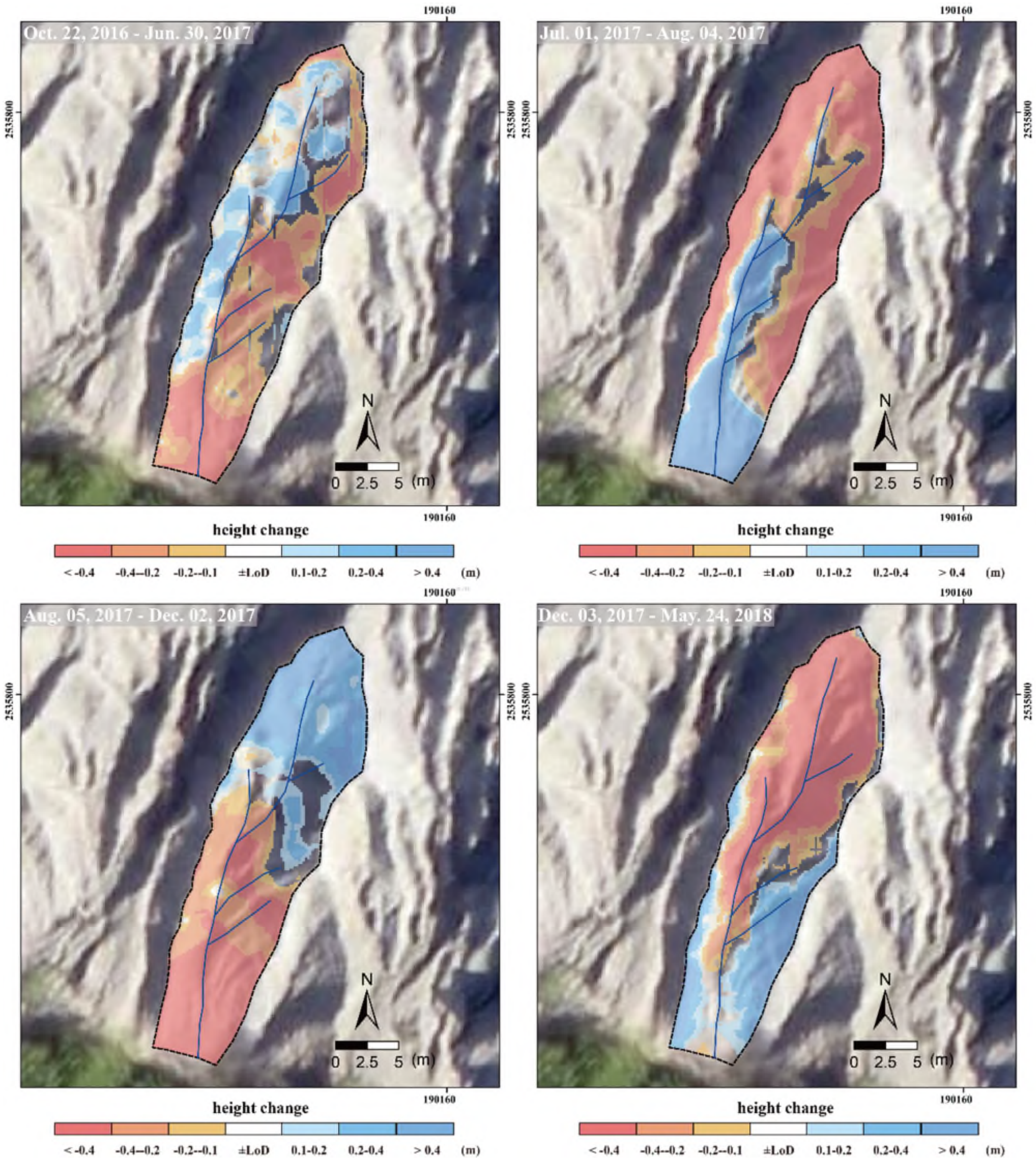

Supplementary Figure 4-29 Distribution of height change obtained from the DEMs by the UAV survey. Blue lines denote drainages. The ortho-images of hillslope were conducted on Oct. 22, 2016 by UAVs survey of this study (see Methods).

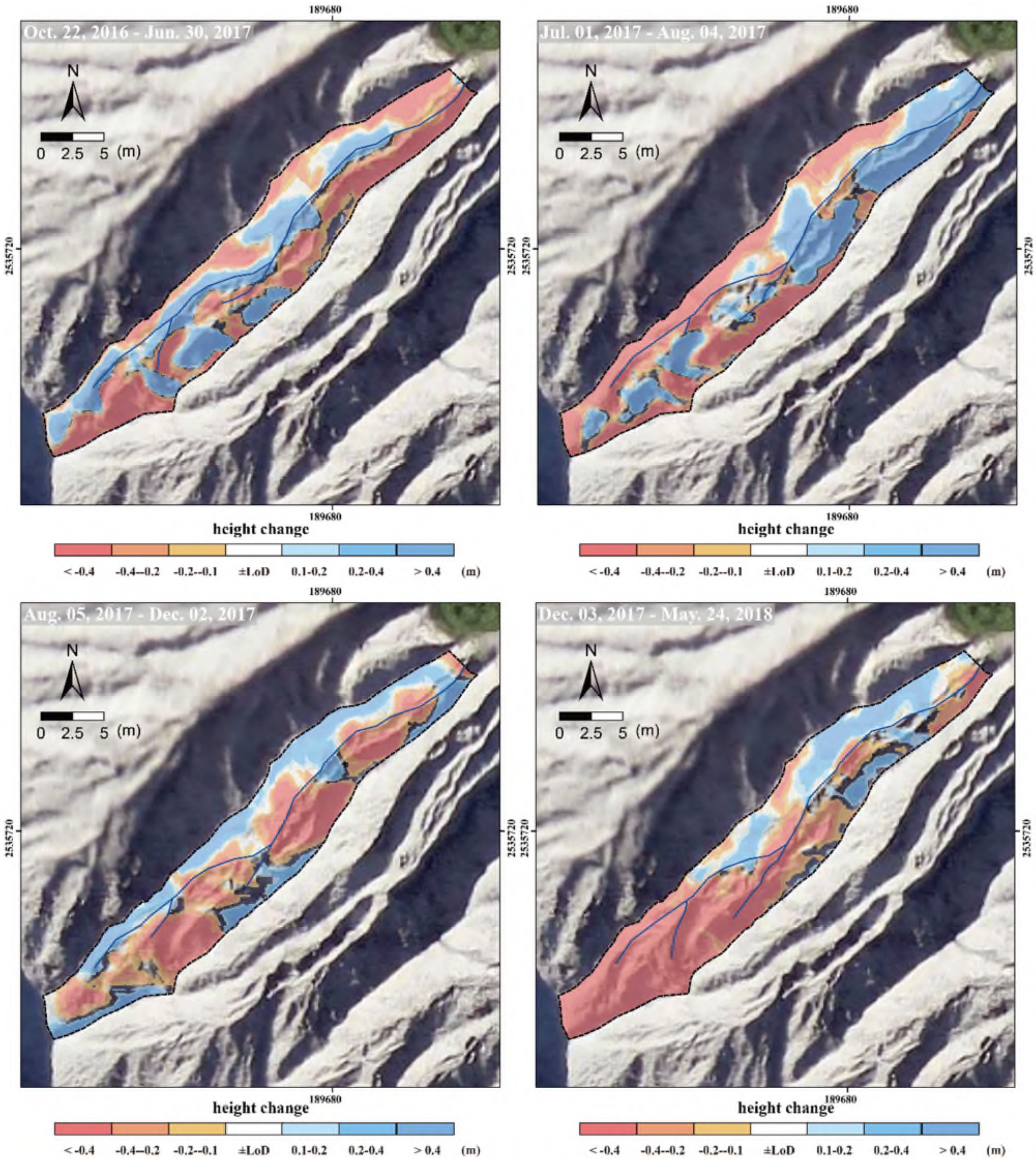

Supplementary Figure 4-30 Distribution of height change obtained from the DEMs by the UAV survey. Blue lines denote drainages. The ortho-images of hillslope were conducted on Oct. 22, 2016 by UAVs survey of this study (see Methods).

Survey uncertainties

The survey error of UAV-SfM-derived DEMs can be divided into three types:

The first source of uncertainty (Type-A) is due to survey errors associated with the position of ground control points during field work. We used e-GPS (see method) to measure the spatial coordinates and elevation of 11 control points within the study area. Errors in the e-GPS measurements can be caused by atmospheric interference, instrument system error and human operation. To have a means to check for survey quality, we defined the 9th ground control point as the origin of coordinate system, and used a total station (LEICA TS02) to measure the positions of eight other ground control points using the 9th point as the base location. The remaining two ground control points did not have a direct line of sight to the base location. This procedure allows to obtain a control point location error estimate, assuming that the accuracy of the total station is higher than that of the e-GPS. The results of the comparison of the two survey methods is shown in Supplementary Table 1. The difference is 1 ± 1.3 cm in the horizontal and 1.4 ± 2.8 cm in the vertical.

The second source of uncertainty (Type-B) is due to errors in locating ground control points in the drone images. Each ground control point is captured by multiple images from the same survey, and therefore position of the ground control point in the image is subject to errors of projection and image deformation. The Acute3D software provides quality reports for the UAV surveys. These are summarized in Supplementary Table 2. The RMS deviation varies between 0.6 cm to 3.6 cm in the horizontal direction and 0.2 cm to 0.6 cm in the vertical.

The third source of uncertainty (Type-C) is due to the position uncertainty of checkpoints. The image processing produces image distortion and affects the authenticity of the DSMs. Therefore, to quantify the degree of image distortion, the Acute3D software establishes evenly distributed checkpoints on orthophotos and calculates the position uncertainty of these checkpoints (see Supplementary Figure 6). The position uncertainty of checkpoints is shown in Supplementary Table 2. The mean error varies from 2.0 cm to 13.0 cm in the horizontal direction and 0.7 cm to 5.6 cm in the vertical. Overall, the errors in the vertical direction is the level of millimeters to centimeters, with few locally larger or smaller errors. As such, the mean errors give a good representation of the overall errors. In addition, errors show circular symmetry, indicating that they capture random rather than systematic errors.

In summary, the main source of error results from Type-C, image distortion in areas without control points.

We use the elevation data for two main purposes, the detection of erosion or deposition, and the calculation of local slope and its change.

In the first application, each pixel is looked at individually. As long as the same pixel refers to the same area in subsequent surveys, the vertical uncertainty is relevant only for change detection of elevation. Image distortion may introduce an effect of the horizontal error, which scales linearly with slope. That is, if a pixel in the later survey contains area that was attributed to different pixels in the earlier survey, the shift in the coordinate system may lead to an apparent change in elevation that is proportional to the local slope times the horizontal error.

In the second application, slope is calculated as the ratio of elevation differences of adjacent pixels

and pixel size. Assuming normally distributed errors, the relative error in slope can be evaluated as the square root of the sum of the squares of the relative horizontal and vertical errors. For slope changes, individual pixels are compared and similar considerations apply as given above for the errors in elevation changes.

From the brief overview, it is clear that both horizontal and vertical errors can contribute to the errors in the quantities of interest. Given that the relative errors may be substantial (up to >40% of the pixel size for the horizontal error and similar mean vertical relative errors for locations), the concerns of the reviewers are reasonable. We want to make a few points to justify our approach for analyzing the data.

First, we note that both horizontal and vertical errors can be expected to be highly spatially correlated. For example, if a given pixel has an error due to distortion, in the horizontal or vertical, we expect adjacent pixels to have an error of similar magnitude and direction. This spatial dependence of the uncertainty patterns can be expected to strongly decrease the uncertainties relevant for the analysis, especially for the calculation of slope, which utitlizes data from adjacent pixels. Unfortunately, the degree of spatial dependence cannot be quantified with the available data.

Second, due to the high-resolution topography, each dataset consists of a large number of data points. Over the entire survey area, we have analyzed more than 1 million individual data points. Due to the law of large numbers, according to which the uncertainty of individual statistics scales with the inverse of the square root of the total number of data points, the central statistics that we use for our interpretation are very robust.

Third, the reliability of the height change involves data from multiple periods, and therefore we use LOD (Wheaton et al., 2003) to filter out the data with excessive height differences.

Fourth, the availability of large numbers of data points allows the rigorous comparison of distributions, which also allows observing differences for the entire ensembles of data, rather than individual data points or central statistics.

In light of the discussion of uncertainties above, we approached the data analysis with three broad strategies.

First, we considered central statistics, mainly the median, and changes therein, since, given the large number of data points available in the surveys, these bear small uncertainties.

Second, we used the Kolmogorov-Smirnov test (KS test) to assess differences in the distributions of the data. The test shows that all of period significantly different from all other periods at the 5% significance level (see Fig. 4 and Supplementary Table 3).

Third, we used binning approaches to assess central tendencies in the data. This method is advantageous, as it retains the diminishing errors due to the law of large numbers, but allows to assess trends with potential forcing variables. We note here that the correspondence of trends for example of gradient change for the different survey episodes and the changes due to typhoon Morakot is a strong indication that we do not observe a statistical fluke.

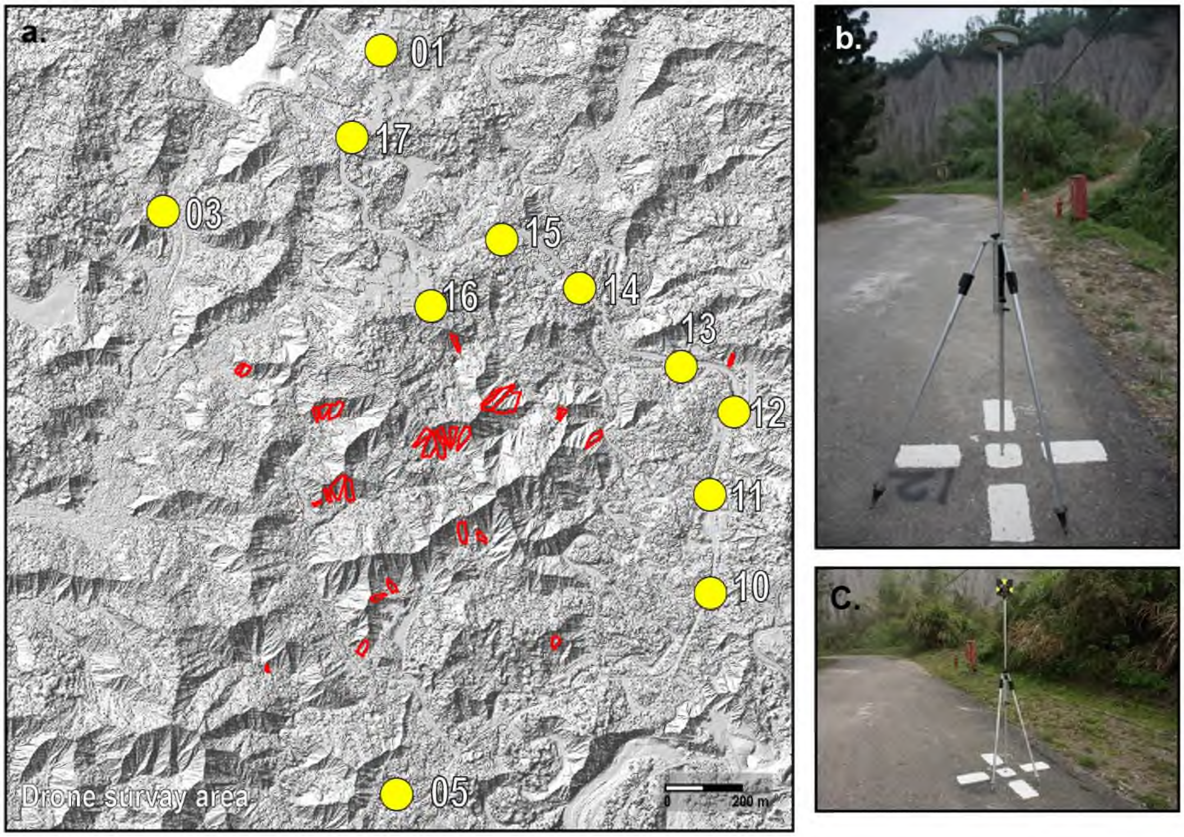

Supplementary Figure 5 Distribution of ground control points of DEMs. a The yellow dots denote the ground control points and the white numbers denote identities. red areas denote the target slopes **b** On-site measurement of e-GPS. **c** On-site measurement of total station (in panel c, which is the prism of total station).

Supplementary Table 1 Uncertainty estimates from comparison of total station and e-GPS surveys of the ground control points.

ID	E-GPS			Total station			Difference of E-GPS and Total station	
	Lon.	Lat.	Height (m)	Lon.	Lat.	Height (m)	Horizontal-axis (m)	Vertical -axis (m)
01	120.410	22.928	125.927	-	-	-	-	-
03	120.405	22.925	71.266	-	-	-	-	-
05	120.410	22.914	75.424	120.410	22.914	75.456	0.043	0.032
10	120.416	22.918	82.125	120.416	22.918	82.165	0.002	0.040
11*	120.416	22.920	80.473	120.416	22.920	80.473	0.000	0.000
12	120.417	22.921	80.704	120.417	22.921	80.687	0.000	-0.017
13	120.416	22.922	78.859	120.416	22.922	78.839	0.003	-0.020
14	120.414	22.924	92.087	120.414	22.924	92.078	0.011	-0.009
15	120.412	22.924	107.070	120.412	22.924	107.078	0.011	0.008
16	120.411	22.923	128.058	120.411	22.923	128.094	0.018	0.036
17	120.409	22.926	126.007	120.409	22.926	126.067	0.004	0.060
Mean (m)							0.010	0.014
Standard deviation (m)							0.013	0.028

Supplementary Table 2 Specification of the five unmanned aerial vehicle survey.

Survey date	GSD (cm/pixel)	control points errors		position uncertainty of checkpoints		
		RMS of horizontal errors (m)	RMS of vertical errors (m)	X-axis mean error (m)	Y-axis mean error (m)	Z-axis mean error. (m)
Oct.22, 2016	11.0	0.036	0.006	0.0250	0.0200	0.0072
Jul. 01, 2017	11.3	0.006	0.003	0.0850	0.0690	0.0330
Aug. 05, 2017	11.1	0.011	0.002	0.0777	0.0629	0.0289
Dec. 03, 2017	9.7	0.031	0.004	0.1297	0.1137	0.0562
May 24, 2018	11.3	0.030	0.006	0.1281	0.1035	0.0484

Supplementary Table 3 Statistical test of gradient distribution of all epochs.

T-test for badland data set

		Observed					Simulated			
		1	2	3	4	5	1	2	3	4
Observed	1	-	-	-	-	-	-	-	-	-
	2	2.03e-33*	-	-	-	-	-	-	-	-
	3	0*	0*	-	-	-	-	-	-	-
	4	0*	0*	1.03e-96*	-	-	-	-	-	-
	5	1.48e-253*	7.45e-131*	0*	2.15e-110*	-	-	-	-	-
Simulated	1	6.53e-65*	1.64e-25*	4.97e-92*	2.74e-32*	0.41	-	-	-	-
	2	0*	0*	1.02e-128*	5.80e-276*	0*	6.29e-178*	-	-	-
	3	2.43e-87*	1.54e-36*	1.83e-80*	5.03e-24*	0.32	0.19	1.26e-202*	-	-
	4	6.05e-46*	1.09e-14*	1.88e-119*	4.32e-50*	5.46e-04*	0.08	2.64e-204*	0.001*	-

T-test for typhoon Morakot data set

	Pre-typhoon Morakot
Post-typhoon Morakot	0*

Ks-test for badland data set

		Observed					Simulated			
		1	2	3	4	5	1	2	3	4
Observed	1	-	-	-	-	-	-	-	-	-
	2	9.73e-69*	-	-	-	-	-	-	-	-
	3	0*	0*	-	-	-	-	-	-	-
	4	0*	0*	3.36e-160*	-	-	-	-	-	-
	5	2.19e-236*	6.19e-101*	0*	2.84e-296*	-	-	-	-	-
Simulated	1	1.48e-159*	8.85e-79*	2.13e-141*	9.38e-163*	3.73e-34*	-	-	-	-
	2	0*	0*	4.31e-160*	0*	0*	1.19e-134*	-	-	-
	3	1.31e-135*	7.46e-65*	5.97e-55*	1.83e-54*	7.40e-17*	2.18e-18*	3.41e-143*	-	-
	4	1.35e-116*	5.90e-48*	2.19e-151*	1.40e-166*	1.37e-32*	0.001*	8.68e-155*	1.17e-18*	-

Ks-test for typhoon Morakot data set

	Pre-typhoon Morakot
Post-typhoon Morakot	0*

* can be considered as significant difference at the 5% level.

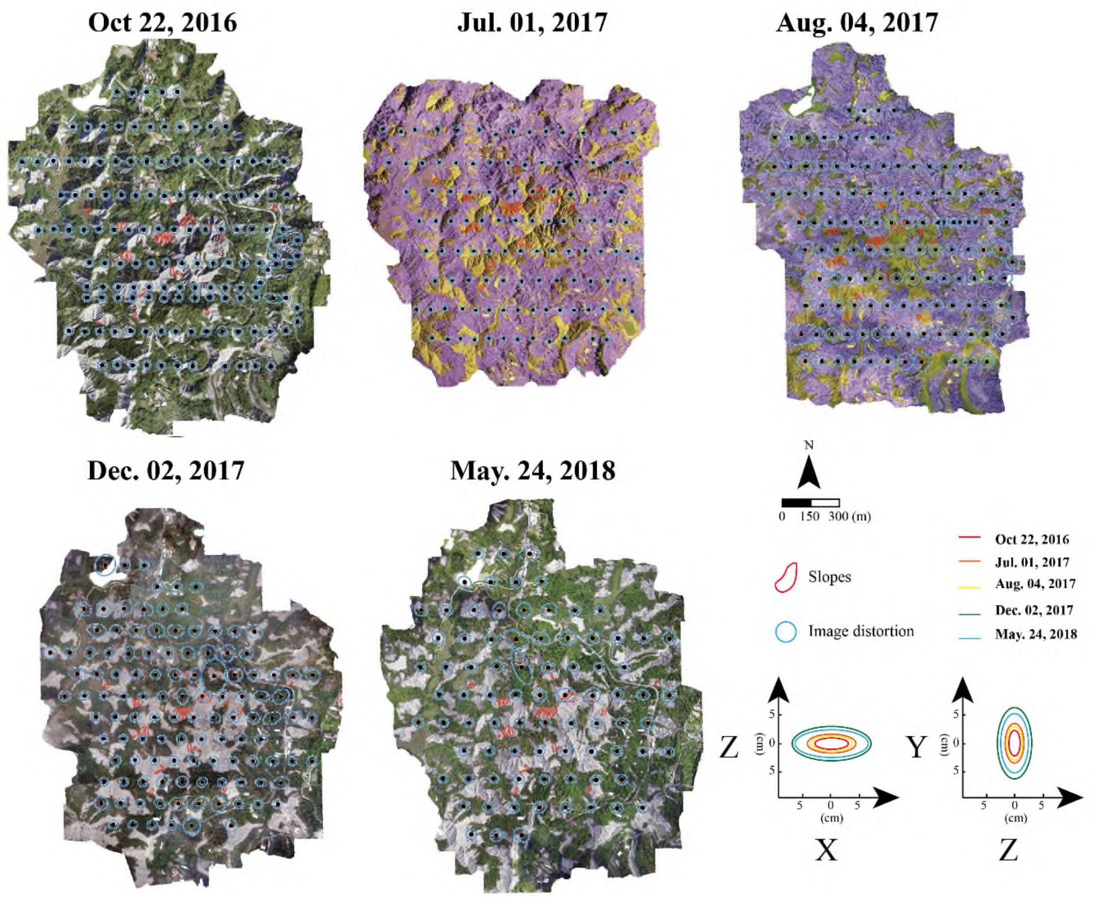

Supplementary Figure 6 The results of checkpoints of position uncertainity. Black dots denote check points and bule circles denote uncertainity. The inset plots show the position uncertainty of checkpoints on X-Z plane and Y-Z plane, respectively, colors are corresponded to survey date.

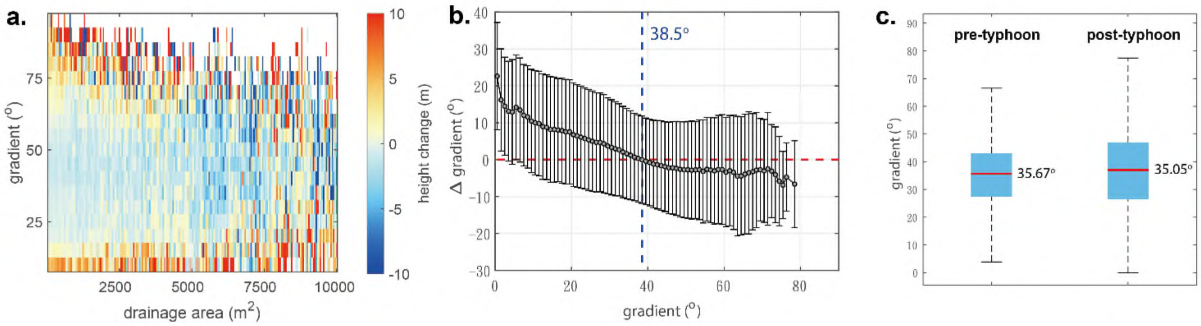

Supplementary Figure 7 Related topographic change data due to typhoon Morakot in upstream of Lin-Bain catchment. a Distribution of mean spatiotemporal erosion for the survey periods. Color indicates height change, warm and cold colors represent positive and negative values. **b** Gradient change as function of hillslope gradient. **c** Distribution of hillslope gradients..

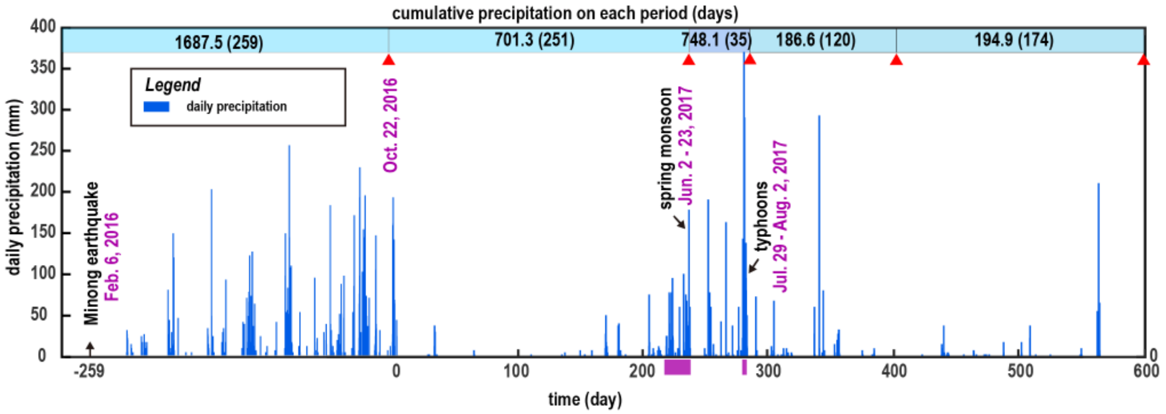

Supplementary Figure 8 Daily precipitation between Minong earthquake and end of survey date. Daily and cumulative precipitation from hourly measurements, red triangles mean UAV survey date.

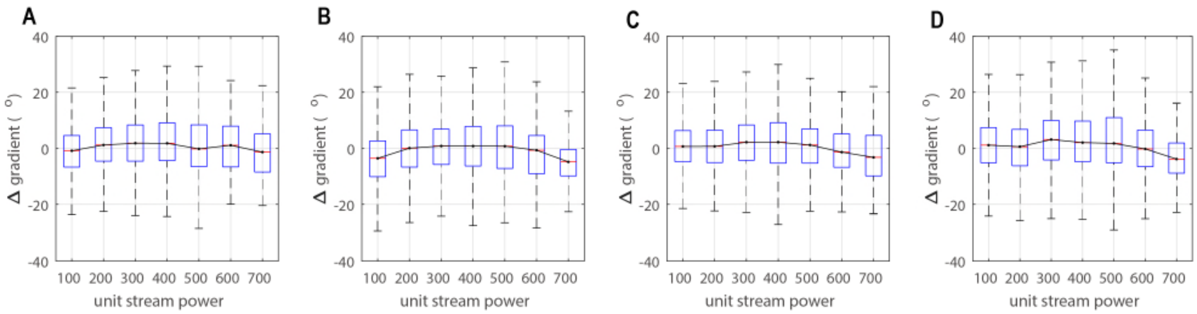

Supplementary Figure 9 Change in gradient as a function of unit stream power. **A** Oct. 22, 2016–Jun. 30, 2017 (monsoon). **B** Jul. 01, 2017–Aug. 04, 2017 (typhoons). **C** Aug. 05, 2017–Dec. 02, 2017 (winter precipitation). **D** Dec. 03, 2017–May 25, 2018 (winter precipitation).
